# The Tsunami Threat to Sydney Harbour, Australia: Modelling potential and historic events

**DOI:** 10.1038/s41598-018-33156-w

**Published:** 2018-10-15

**Authors:** Kaya M. Wilson, Stewart C. R. Allen, Hannah E. Power

**Affiliations:** 1 School of Environmental and Life Sciences, The University of Newcastle, Callaghan, Australia; 2 Bureau of Meteorology, Melbourne, Australia

## Abstract

Tsunami modelling of potential and historic events in Australia’s Sydney Harbour quantifies the potentially damaging impacts of an earthquake generated tsunami. As a drowned river valley estuary exposed to distant source zones, these impacts are predominantly high current speeds (>2 m/s), wave amplification and rapid changes in water level. Significant land inundation only occurs for scenarios modelled with the largest waves (9.0 M_W_ source). The degree of exposure to the open ocean and the geomorphology of locations within the Harbour determine the relative level of these impacts. Narrow, shallow channels, even those sheltered from the open ocean, create a bottleneck effect and experience the highest relative current speeds as well as elevated water levels. The largest maximum water levels (>8 m) occur in exposed, funnel-shaped bays and wave amplification is greatest at locations exposed to the open ocean: >7 times deep water wave heights for 9.0 M_W_ source waves. Upstream attenuation rates of runup and maximum water level show a linear correlation with wave height parameters at the 100 m depth contour and may provide some predictive capabilities for potential tsunami impacts at analogous locations. In the event of a tsunami in Sydney Harbour, impacts may threaten marine traffic and infrastructure.

## Introduction

Tsunami are one of the key natural hazards that threaten coasts globally. Tsunami impacts range from those only detectable by instruments to those, such as the Indian Ocean tsunami of 2004, which devastated communities across an entire region and was the deadliest tsunami in recorded history^1^. The impacts of tsunami are known to vary for different coastal morphological environments. Estuaries, such as the Vellar estuary in India^2^, along with estuaries in Chile^3^ and Japan^4–6^, have been observed to experience tsunami inundation many times that of the adjacent open ocean coastline, as the estuary facilitates tsunami propagation further inland. Estuaries are also known to experience wave height increases and modifications^7^ as a result of the tsunami waves interacting with the bathymetry^8,9^ and bay shape^10^. For estuaries with complex geomorphology, such as Australia’s Sydney Harbour, detailed tsunami modelling is the most accurate way to determine potential tsunami impact.

**Figure 1:**
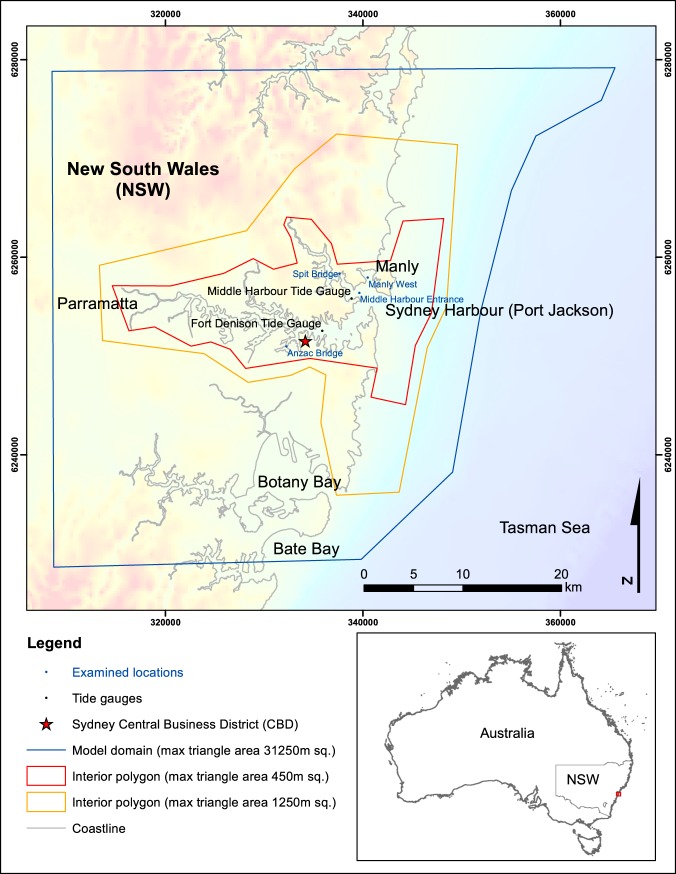
Study site and locations selected to represent potential tsunami impact within the context of the model domain. Grid coordinates are provided in WGS84 UTM zone 56S. Image created by KMW using ESRI ArcMap 10.3.1 http://www.esri.com/arcgis/about-arcgis with elevation (https://ecat.ga.gov.au/geonetwork/srv/eng/search#!a05f7892-fae9-7506-e044-00144fdd4fa6) and coastline data (https://ecat.ga.gov.au/geonetwork/srv/eng/search#!a05f7892-eae3-7506-e044-00144fdd4fa6) from © Commonwealth of Australia (Geoscience Australia) 2017.

The city of Sydney in New South Wales (NSW), Australia’s most populated city, is centred on the picturesque Sydney Harbour (Port Jackson; Fig. 1). Sydney Harbour is both a working port and major location for recreational boating and leisure with large numbers of people on and around the water every day. There is geological and historical evidence for tsunami affecting Sydney and the NSW coastline^11^ and the NSW State Emergency Service (SES) Tsunami Emergency Sub Plan estimates a large tsunami with the potential to impact the entire NSW coast would directly threaten between 250,000 and 1.5 million people^12^.

Although the tsunami hazard level for NSW is considered moderate^13,14^, it is present. One of the most catalogued historic tsunami events to affect Sydney Harbour is the 9.5 M_W_ Chilean tsunami of May 1960^15^. This tsunami was caused by a subduction zone earthquake, as are the majority of tsunami globally (~73%)^16^. Accounts from the 1960 Chilean event include reports of unusually large wave heights, extreme current speeds, and rapid changes in water level. The direct impacts of this event included dragged and broken moorings, vessels being swept into bridges and wharfs, as well as significant scouring and localised coastal erosion^15^. In contrast, the 2011 9.1 M_W_ Tohoku Japanese tsunami was recorded by Sydney Harbour tide gauges but no damage was reported for this event^17^.

The geomorphological shapes of estuaries can result in amplified tsunami impacts even during minor events. For example, strong currents caused by tsunami, have been known to cause significant damage in estuaries even when little or no runup or inundation has occurred^15,18^. In California, current speeds of approximately 4.5 m/s resulting from the 2011 Japanese tsunami caused over US$50 million of damage in harbours across the state despite warnings being issued. The tsunami arrived in California at low tide and so these high current speeds were coupled with very little inundation above the average high tide level^18^. Reports of tsunami generated current induced damage in estuarine ports and harbours have included ships being torn from moorings and subsequent groundings or impact^15,19–21^. In instances when warnings have been given and vessels were evacuated from harbours or restricted from entering harbours, less damage occurred^18^.

An additional tsunami generated hazard that has been shown to be particularly damaging to estuarine ports and harbours includes depths outside of the normal tidal range. Abnormally low minimum depths, abnormally high maximum depths, and rapid changes in depth caused by water movement during a tsunami are a hazard to estuary users and, in particular, to vessel navigation. Issues can arise where under keel clearance may be at a premium^22^ and/or vessel traffic requires passage underneath low bridges. The morphological changes caused by the erosion and deposition of sediment can also alter depths significantly, with depths remaining dramatically altered after the tsunami event is over^12,18,23^. For locations such as Sydney Harbour, some distance from the nearest source zones and less likely to experience extreme inundation, high current speeds and depth variation may be the dominant impact of a tsunami.

Previous research has shown that tsunami wave trains and the tide interact in a non-linear manner in estuaries^24–26^. The major cause of the tsunami-tide interaction has been demonstrated to be tidally induced ocean depth changes^24^. It has been shown that the tide-tsunami interaction can result in either intensification or damping of cumulative tsunami-tide impacts, depending on mean basin depth^24^. Changes in depth due to the ocean tide, alter the tsunami propagation, amplification, and dissipation, thus it is crucial in modelling studies that the tide and tsunami are appropriately coupled to reduce the limitations of the model^24–26^.

The Joint Australian Tsunami Warning Centre (JATWC) has completed rupture and deep water propagation modelling of numerous tsunami scenarios in order to create a tsunami scenario database for Australia: T2. The T2 database includes tsunami scenarios generated by earthquakes of 7.0, 7.5, 8.0, 8.5 and 9.0 M_W_ from source zones relevant to Australia and allows these tsunami scenarios to be extracted at any deep water location^27^. Shallow water inundation modelling is then required to estimate the potential coastal and terrestrial impacts of these tsunami scenarios.

Previous shallow water modelling for the Sydney region includes the tsunami modelling of Botany and Bate Bay, two estuaries to the south of Sydney, (see Fig. 1) by Dall’Osso *et al*.^28^. Dall’Osso *et al.*^28^ used static tides and tsunami scenarios with Average Recurrence Intervals (ARI) of 100, 1,000 and 10,000 years to investigate maximum inundation extents and maximum flow velocities. Their research also indicated the number of buildings that would potentially experience inundation. Another larger study, by Cardno^14,29^ also completed modelling for Botany Bay as well as coarse modelling of the outer portion of Sydney Harbour with a detailed focus on Manly. The work by Cardno modelled tsunami with dynamic tides^14,29^ and showed the vulnerability of Manly to inundation. The Cardno study also identified the areas of Botany Bay susceptible to inundation from scenarios with ARI ranging from 200 to 10,000 years. The Cardno scenarios were sourced from Kermadec, New Hebrides, Puysegur, South Chile and Tonga trenches.

Previous efforts have also been made to develop attenuation rules for some types of estuaries. Leonard *et al*.^30^ created a GIS based attenuation rule for maximum water level of tsunami propagation up rivers in Samoa. This was validated to some extent in the same paper and later against the East Japan (Tohoku) tsunami of 2011 by Fraser & Power^31^ with a greater focus on maximum potential runup. This rule describes a 0.25% maximum water level attenuation up-river (*i.e.* 1 m water level attenuation for every 400 m of distance travelled up-river). The derived attenuation rule for maximum potential runup uses a specified and calculable maximum potential runup at the coast, then follows the same attenuation rule as maximum water level attenuation, with an additional rule of 1 m maximum runup attenuation for every 50 m travelled across land from the river banks^31^.

Sydney Harbour is classed as a drowned river valley estuary under the Roy *et al*.^32^ morphological classification system. Drowned river valleys typically occur in deeply incised bedrock valleys, opening into a semi-protected bay^32^. Although comprehensive modelling provides the most reliable estimate of potential tsunami impacts, it is not always possible. The observation of rules or tsunami impact patterns can help provide first pass estimates of potential tsunami impacts for comparable locations where modelling is not possible as well as help further our understanding of tsunami behaviour.

To our knowledge, this paper presents the first validated, high-resolution tsunami inundation model for the full extent of Sydney Harbour (Port Jackson), from the estuary mouth to Parramatta (see Fig. 1). In this study we provide a comprehensive account of the potential tsunami impacts for this iconic estuary, suggest attenuation patterns and highlight the geomorphological features that show the most vulnerability to tsunami impacts. We present these results with a view to deepening understanding of tsunami impacts in drowned river valleys with distant source zones.

## Results

Here we use the hydrodynamic model ANUGA to simulate tsunami inundation in Sydney Harbour for a range of potential and historic scenarios (Table 1). ANUGA solves the non-linear shallow water wave equations to describe tsunami behaviour at each node in an unstructured triangular elevation mesh and for each time step over a specified time period. These calculations were completed for every scenario modelled and results were extracted to describe terrestrial inundation, flow velocities, water levels and wave heights across the study site. Scenarios are named according to the source zone, the magnitude of earthquake from which the tsunami was modelled, and the tidal stage the tsunami was modelled to coincide with (Table 1). For example, NH90high represents a wave generated by a 9.0 M_W_ earthquake at the New Hebrides trench that is modelled such that the maximum wave in the tsunami wave train is coincident with the peak of the high tide.

**Table 1:** Details of all tsunami scenarios modelled. PTHA estimated ARI represents the Annual Recurrence Interval as estimated by the Probabilistic Tsunami Hazard Analysis of Australia (Burbidge *et al*.^33^).

Wave Train Name	T2 Reference Number	Source Zone	T2 Source Earthquake Magnitude M_W_	PTHA estimated ARI	Input wave parameters at the 100 m depth contour model boundary				
					*H*_*rms* (m)_	*H*_*max* (m)_	*H*_*stdev* (m)_	*Mean T* (sec)	Amplitude above still water level (m)
**T2 Scenarios**									
P75	T2_214a_ts	Puysegur	7.5	25	0.01	0.05	0.01	2016	0.02
P80	T2_213b_ts	Puysegur	8.0	50	0.05	0.18	0.04	2074	0.13
P85	T2_217c_ts	Puysegur	8.5	200	0.17	0.53	0.14	2858	0.36
P90	T2_216d_ts	Puysegur	9.0	4700	0.44	1.36	0.34	3297	1.33
NH80	T2_198b_ts	New Hebrides	8.0	30	0.04	0.14	0.03	2178	0.06
NH85	T2_197c_ts	New Hebrides	8.5	110	0.16	0.48	0.13	2800	0.26
NH90	T2_194d_ts	New Hebrides	9.0	550	0.37	1.00	0.29	3000	0.61
**Historic Events**									
Chi1960	T2_401d_ts (scaled)	Chile	9.2	71	0.10	0.40	0.07	7905	0.18
Toh2011	T2_311c_ts (scaled)	Japan	9.0	36	0.05	0.14	0.03	4029	0.08

It should be noted that this is a statistical relationship that may resolve better for wave trains with larger ARI. Where wave height is measured from peak to trough, wave parameters are defined as: root mean squared of all wave heights (*H*_*rms*_), maximum wave height (*H*_*max*_), standard deviation of all wave heights (*H*_*std*_) and mean wave period (*Mean* *T*). Wave trains in the text are suffixed with ‘high’, ‘low’ and ‘historic’ to describe tide-tsunami phasing *e.g.* P75low represents wave train P75 modelled for peak tsunami waves to coincide with a low tide, and Chi1960historic represents wave train Chi1960 summed to the historic tide at that occurred during the event. Tsunami scenarios are obtained from the T2 database^27^.

Further details of all tsunami sources are provided in Methods: Tsunami Wave Train Boundary Conditions.

The Probabilistic Tsunami Hazard Analysis of Australia (PTHA)^33^ provides the numerical relationship between maximum wave amplitude of a tsunami wave train with Average Recurrence Intervals (ARI) for locations across Australia and in this case for Sydney Harbour. Using the data from the PTHA, the ARI of the potential tsunami scenarios modelled were estimated to be 100–4700 for those sourced from earthquakes ≥8.5 M_W_, and 25–50 for those of ≤8.0 M_W_ (Table 1). It should be noted that this is a statistical relationship that may resolve better for wave trains with larger ARI.

### Maximum Inundation Extent

**Figure 2:**
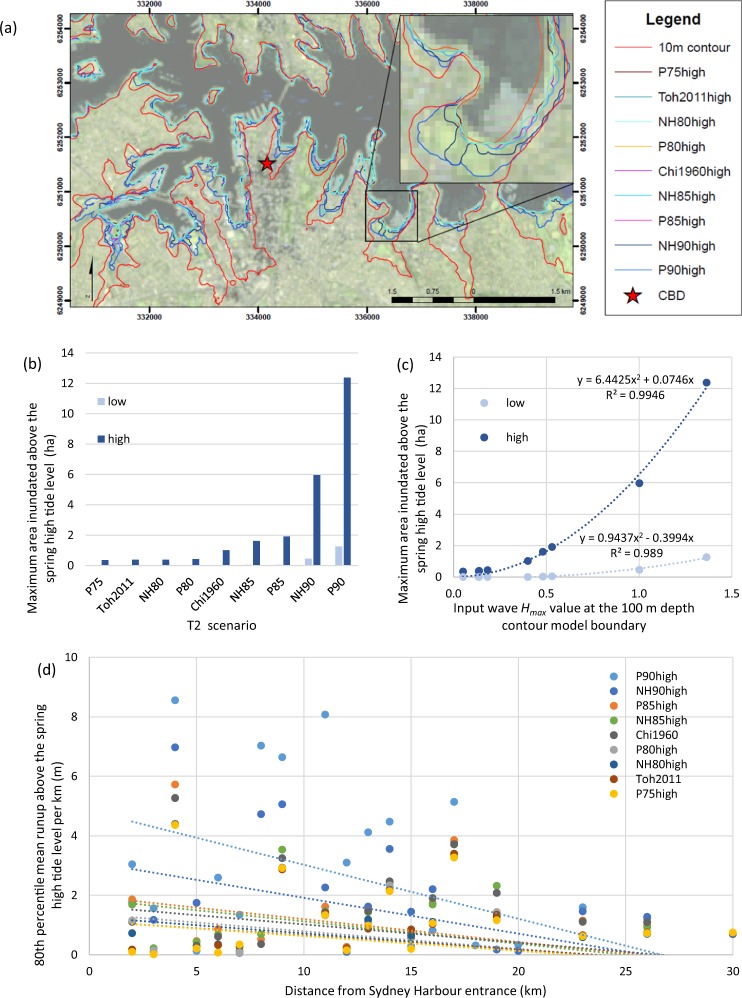
Maximum inundation extent, maximum inundation area and 80th percentile mean runup. (**a**) Maximum inundation extents for tsunami scenarios coinciding with a high tide with Rushcutters Bay shown in detail in the inset. The 10 m elevation contour is provided for reference and the red star represents the Sydney Central Business District (CBD) as per Fig. 1. Grid coordinates are provided in WGS84 UTM zone 56S. Image created by KMW using ESRI ArcMap 10.3.1 http://www.esri.com/arcgis/about-arcgis, coastline data (https://ecat.ga.gov.au/geonetwork/srv/eng/search#!a05f7892-eae3-7506-e044-00144fdd4fa6) from © Commonwealth of Australia (Geoscience Australia) 2017 and satellite imagery from the USGS landsat collection (https://earthexplorer.usgs.gov/). (**b**) Maximum area inundated above the high tide level for all scenarios modelled. It should be noted that due to the constraints on mesh resolutions, areas with minor inundation are not well resolved. The legend indicates model scenarios, which were modelled for peak waves to coincide with a ‘low’ or ‘high’ tide. (**c**) The correlation between the input scenario *H*_*max*_ value at the 100 m depth contour model boundary condition and the maximum area inundated. The legend indicates which scenarios were modelled for peak waves to coincide with a ‘low’ or ‘high’ tide. (**d**) The mean of the 80^th^ percentile maximum runup values above the spring high tide level, per km. The distance from Sydney Harbour entrance is provided on the ‘x’ axis. Data shown represents the south side of the Harbour.

**Figure 3:**
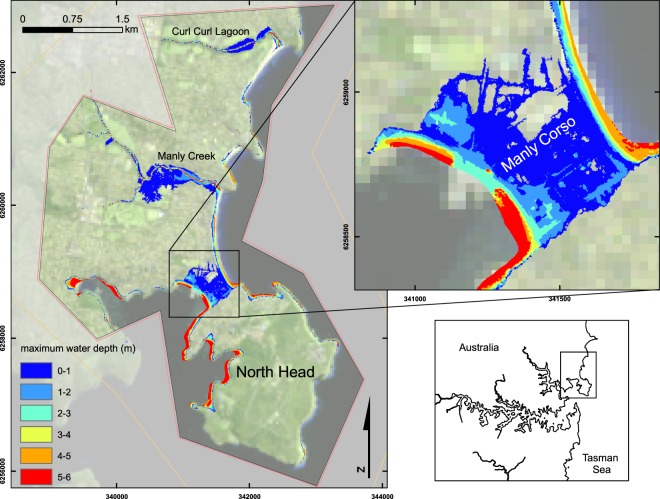
Maximum inundation extent and inundation depths for tsunami scenario P90high for the high resolution Manly model (maximum triangle area of 2 m^2^). Depths are given relative to MSL and grid coordinates are provided in WGS84 UTM zone 56S. Image created by KMW using ESRI ArcMap 10.3.1 (http://www.esri.com/arcgis/about-arcgis), coastline data (https://ecat.ga.gov.au/geonetwork/srv/eng/search#!a05f7892-eae3-7506-e044-00144fdd4fa6) from © Commonwealth of Australia (Geoscience Australia) 2017 and satellite imagery from the USGS landsat collection (https://earthexplorer.usgs.gov/).

Terrestrial inundation increases with increasing earthquake magnitude, with significant inundation limited to the highest magnitude events modelled. The majority of inundation occurs in the low-lying embayments along the southern side of the Harbour (Fig. 2a) and across the low lying isthmus connecting North Head to Northern Sydney in Manly (Fig. 3 and see Fig. 1 for location). The two scenarios with the largest waves, NH90high and P90high, both stand out as having maximum inundation extents significantly greater than other scenarios modelled (Fig. 2b). Historic events modelled with historic tides showed inundation too minimal to be visible in Fig. 2: Toh2011historic resulted in 0.006 ha above the high tide level and Chi1960historic, 0.039 ha.

Maximum inundation extents were shown to be dependent on whether the peak tsunami waves coincided with a low or high tide, using spring tides extracted from observed data in January 2015. All scenarios in which peak tsunami waves coincided with high tide showed some inundation above the spring high tide level, with NH90high (6 ha) and P90high (12 ha) inundating areas 3–4 times greater than that of other high tide scenarios (Fig. 2b). For scenarios where peak waves coincided with low tide, inundation above the spring high tide level is only observed in scenarios NH85low, P85low, NH90low, and P90low (Fig. 2b). Significant inundation only occurred in scenarios with the largest wave heights (NH90low and P90low; Fig. 2b). In all scenarios modelled, inundation is restricted to locations below 10 m elevation (Fig. 2a) and, for the region shown in Fig. 2a, only scenarios NH90high and P90high inundate locations with buildings. Supplementary Figs S1–S7 provide maximum inundation maps for the 9.0 M_W_ and 8.5 M_W_ events as well as the Chi1960high scenario.

Maximum area inundated above the spring high tide level was found to correlate well with the tsunami wave height parameters at the model boundary condition (100 m depth contour). Wave height (*H*) is taken here as the distance between the wave peak and trough, where *H*_*max*_ represents the maximum wave height and *H*_*rms*_, the generalised mean of the wave height (root mean squared) for a wave train. Of the two wave height parameters, *H*_*max*_ showed the highest correlation (Fig. 2c) with R^2^ values of 0.99 for a second order polynomial fit for both low and high tide models. *H*_*rms*_ values were also closely correlated to a 2^nd^ order polynomial fit with R^2^ values of 0.94 for low tide models and 0.96 for high tide models.

Maximum runup represents the maximum elevation of inundation. Figure 2d shows the maximum runup in metres above the spring high tide level for the high tide models. In order to show runup attenuation, the 80^th^ percentile runup values  for every km were averaged and the mean was plotted against distance from the Sydney Harbour entrance. R^2^ values remain under 0.3 for this comparison, however the gradients of the trend lines show a clear linear relationship with *H*_*max*_ at the boundary condition (R^2^ = 0.95). This indicates that runup values may be less predictable, however runup attenuation trends are quite clear and may be applicable at comparable sites.

The narrow isthmus in Manly was shown to be particularly vulnerable to inundation, even for scenarios where tsunami coincided with low tide. The area is exposed to the open ocean as well as the Harbour and inundates from both directions. To further investigate this inundation in more detail, a second model domain was developed to examine the P90high scenario in the Manly area with a maximum cell resolution of 2 m^2^. This high resolution model shows inundation across the entire isthmus (with inundation depths below 2 m), potentially isolating North Head (Fig. 3). Inundation depths of over 3 m are limited to within ~50 m of the shoreline, with depths of 2–3 m only occurring in isolated pockets. Main streets, such as Manly Corso, do not inundate beyond 1 m. Significant inundation is also shown to occur at both Manly Creek and Curl Curl Lagoon to the north of the isthmus (Fig. 3).

### Maximum Current Speeds

**Figure 4:**
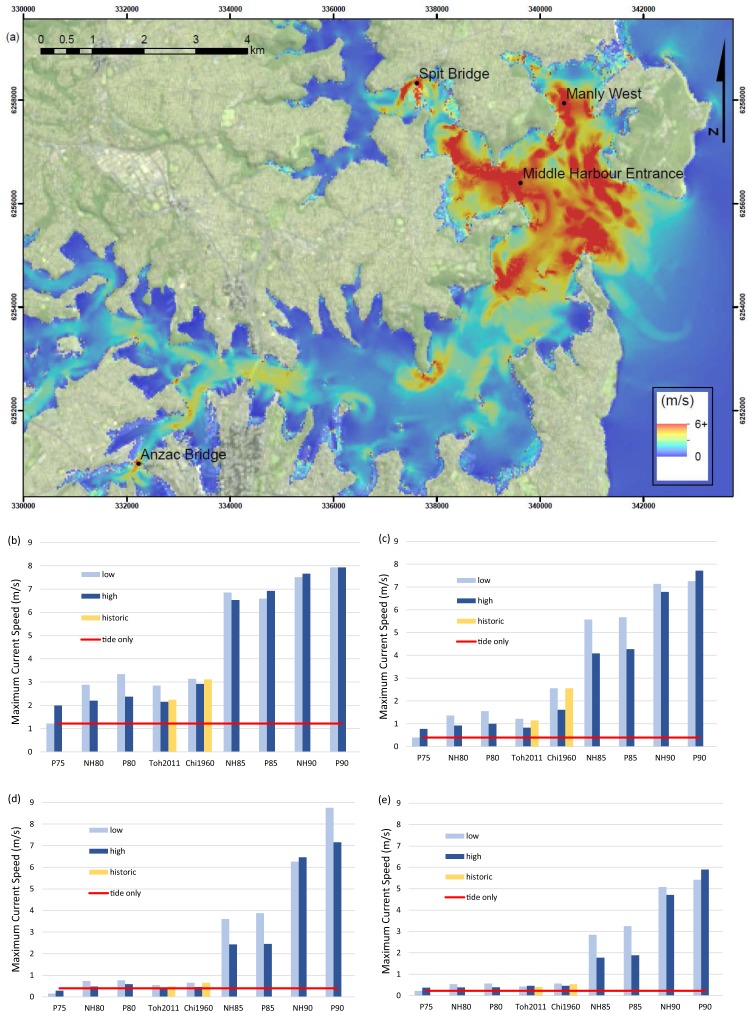
Map of maximum current speeds and graphs of maximum current speeds at four points of interest. (**a**) Sydney Harbour maximum current speed map for scenario P90high. Grid coordinates are provided in WGS84 UTM zone 56S. Image created by KMW using ESRI ArcMap 10.3.1 (http://www.esri.com/arcgis/about-arcgis) and satellite imagery from the USGS landsat collection (https://earthexplorer.usgs.gov/). (**b**) Maximum current speeds for all scenarios modelled at Spit Bridge (Mean Sea Level (MSL) depth = 6.1 m), (**c**) Maximum current speeds for all scenarios modelled at Anzac Bridge (MSL depth = 5.8 m), (**d**) Maximum current speeds for all scenarios modelled at Manly West (MSL depth = 9.2 m) and (**e**) Maximum current speeds for all scenarios modelled at Middle Harbour Entrance (MSL depth = 11.0 m). The ‘tide only’ datum refers to the maximum current speeds for models run with only January 2015 spring tides and no tsunami. The legend specifies if the scenarios were modelled as coincident with low, high or historic tides. Historic tides represent the tide data from the time that a historic tsunami event occurred.

Maximum current speeds are shown to be a significant and widely distributed hazard in the event of a tsunami in Sydney Harbour. Within the Harbour, the highest values for maximum current speeds occur in narrow, shallow, channels such as those where the Spit and Anzac Bridges are located (see Fig. 4). Areas exposed to the open ocean also show high current speeds, relative to open-channel areas that are less exposed. Figure 4a shows the maximum current speed results for P90high in their geographical context. Supplementary Figs S8–S21 show maximum current speeds for all 8.5 M_W_, 9.0 M_W_ and historic scenarios modelled.

Spit Bridge, Anzac Bridge, Manly West, and Middle Harbour Entrance (Fig. 4) were selected as locations known to have high vessel traffic and where preliminary tsunami modelling results showed consistently high current speeds. In all four case study sites, maximum current speeds increase with increasing magnitude of the earthquake source, increasing mean wave period or increasing scenario wave heights. The rate of increase is much greater once the source magnitude of the potential events exceeds 8.0 M_W_ for all four locations (Fig. 4b–e).

Spit Bridge and Anzac Bridge are ~6 and ~11 km upstream and have similar geomorphologies, with narrow, shallow channels that are not directly exposed to the mouth of the Harbour. Both locations showed similar patterns in flow velocities across the scenarios modelled (Fig. 4b,c). Maximum current speeds for scenarios NH85, P85, NH90, and P90 exceed 4 m/s at these two locations for both high and low tide models. Maximum current speeds for scenarios NH80, P80, Toh2011 and Chi1960 exceed 2 m/s at the Spit Bridge for both high, low and historically tided models. At the Anzac Bridge, of these smaller waves, only Chi1960low and Chi1960historic reach maximum current speeds greater than 2 m/s.

Manly West and Middle Harbour Entrance are locations in wide channels, deeper than the Spit and Anzac Bridge locations, and more exposed to the open ocean. Maximum current speeds in both of these locations remain under 1 m/s for scenarios P75, NH80, P80, Toh2011 and Chi1960 but increase to 1.8–3.9 m/s for the 8.5 M_W_ potential scenarios. Maximum current speeds reach 4.7–8.8 m/s for the 9.0 M_W_ potential scenarios (Fig. 4d,e).

The tide phase that the peak tsunami waves coincide with affects the maximum current speeds but not in a consistent manner. For a given earthquake scenario, current speeds were more likely to be greater when the tsunami coincided with low tide than when it coincided with high tide, though exceptions were observed. The differences between the maximum current speeds for corresponding low and high tide scenarios vary, with the greatest differences (1–1.5 m/s) occurring for scenarios NH85 and P85 for all locations except the Spit Bridge (Fig. 4b–e).

### Water Depth Variation: Maximum and Minimum Depths in Locations of Interest

**Figure 5:**
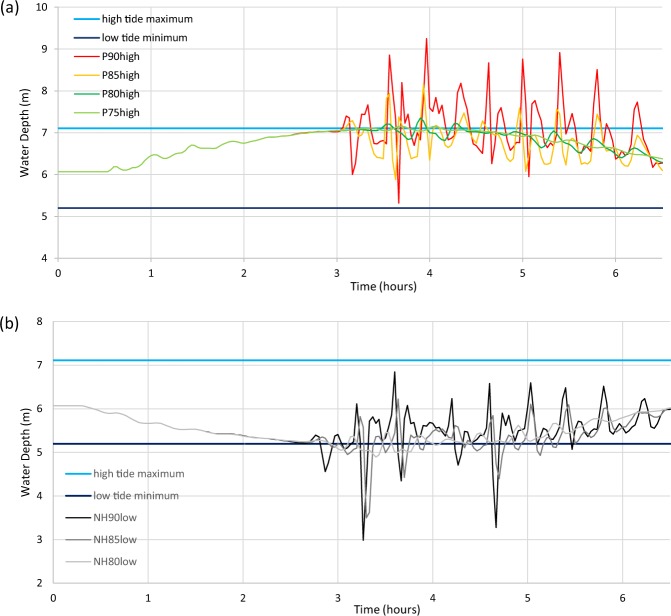
Water depth time series for tsunami scenarios from the Puysegur and New Hebrides trenches at the Spit Bridge location. (**a**) for Puysegur scenarios coincident with high tide (**b**) for New Hebrides scenarios coincident with low tide. Low tide minimum and high tide maximum are the minimum and maximum depths respectively extracted from the tide only model using data from spring tides in January 2015 as specified in Methods: Tide Data. Time is relative to the beginning of boundary condition commencement.

Maximum depths are of most concern for tsunami coincident with a high tide, and minimum depths for tsunami coincident with a low tide. The Spit Bridge, which has vessels moored near it and passing underneath it, has a clearance of ~7 m above Mean Sea Level (MSL)^34^ and is therefore particularly vulnerable to depth hazards. At this location, where the depth at MSL is 6.1 m, the depth range results from the tide only model show a maximum depth of 7.1 m depth at high tide and a minimum of 5.2 m at low tide. This depth range is illustrated in Fig. 5a,b. Figure 5a shows the Puysegur trench scenarios coincident with high tide and Fig. 5b the New Hebrides trench scenarios coincident with low tide.

For tsunami coincident with a high tide, potential scenarios sourced from earthquakes >8.0 M_W_ showed depths exceeding the modelled high tide (spring tides from January 2015) by 0.9–2.1 m, whilst those potential events of ≤8.0 M_W_ exceeded the modelled high tide by 0.05–0.24 m. None of the scenarios modelled across high tide showed minimum depths below low tide levels (Fig. 5a). Maximum and minimum depths should also be considered with respect to wave period as, for this location, depths can range by >3 m in the space of ~30 min (Fig. 5).

For tsunami coincident with low tide, potential tsunami sourced from earthquakes >8.0 M_W_ show minimum depths 1.8–2.2 m shallower than the modelled low tide, whilst those of <8.0 M_W_ show depths 0.7–0.31 m shallower than low tide. Only scenario P90low showed a maximum depth value greater than the modelled high tide level, 0.06 m greater than high tide. Other locations showed a similar pattern across the events modelled, although with different depth range values.

### Wave Heights

#### Maximum Water Level

**Figure 6:**
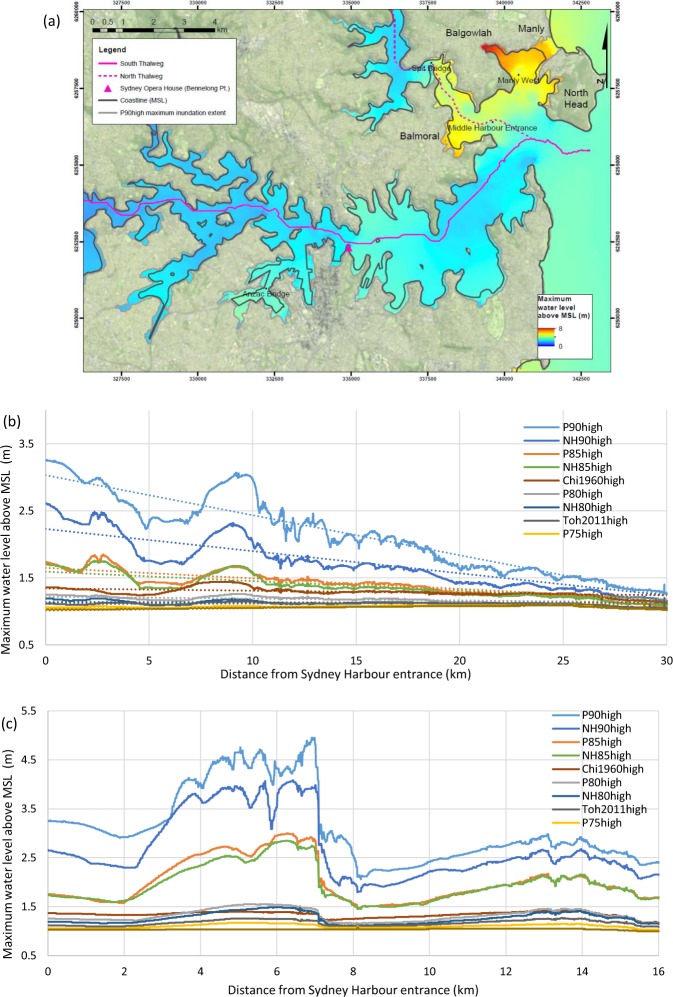
Maximum water level above MSL in Sydney Harbour. (**a**) Maximum water level for P90high in Sydney Harbour. The South and North Thalweg lines are shown in pink. For reference, examined locations Spit Bridge, Anzac Bridge, Manly West and Middle Harbour Entrance are shown as well as the MSL coastline (solid black line), Sydney Opera House (pink triangle) and P90high maximum inundation extent (solid grey line). Grid coordinates are provided in WGS84 UTM zone 56S. Image created by KMW using ESRI ArcMap 10.3.1 http://www.esri.com/arcgis/about-arcgis, coastline data (https://ecat.ga.gov.au/geonetwork/srv/eng/search#!a05f7892-eae3-7506-e044-00144fdd4fa6) from © Commonwealth of Australia (Geoscience Australia) 2017 and satellite imagery from the USGS landsat collection (https://earthexplorer.usgs.gov/). (**b**) South thalweg maximum water level shown against distance from Sydney Harbour entrance (river km). (**c**) North thalweg maximum water level shown against distance from Sydney Harbour entrance (river km).

The maximum water level above MSL that occurs across the P90high tsunami wave train is shown in Fig. 6a as a geographical distribution. Maximum water level is considered in order to show the highest water levels reached during the course of the tsunami event relative to the MSL datum. Maximum wave heights as described in Wave Heights: Maximum Wave Height, are a measure of wave magnitude that is independent of a datum and so does not provide an indicator of water level.

For the P90high event shown in Fig. 6a, maximum water level remains less than 3.5 m above MSL up to 10 km from the Harbour mouth, except for at the coves bordering Balmoral, Balgowlah, Manly, North Head and towards the Spit Bridge, where the maximum water level is markedly higher. The cove at Balmoral reaches maximum levels of >6 m and the cove at Balgowlah reaches >8 m. Embayments further from the mouth of the Harbour, such as those near Anzac Bridge also show higher maximum water levels than those in the main Harbour channel. This distribution pattern is consistent across the events modelled, with the smaller events showing lower absolute maximum water level values but similar overall spatial trends.

 A south and a north thalweg line are mapped in Fig. 6a and the maximum water levels for all scenarios along those lines are plotted in Fig. 6b for the southern thalweg and Fig. 6c for the northern thalweg. Both figures show how a geomorphological narrowing or bottleneck can cause locally elevated water levels, most notably in Fig. 6c at 2–7 km from the Harbour entrance, on the oceanic side of the Spit Bridge location (Fig. 6c). The southern thalweg shows a similar but smaller effect 7–10 km from the Harbour entrance caused by the narrowing of the channel at Sydney Opera House (Bennelong Pt.) (Fig. 6b).

Without the dominant effects of the Spit Bridge bottleneck, the data from the southern thalweg shows that maximum water level attenuations for all scenarios follow an approximately linear trend (e.g. maximum water level attenuation for the scenario P90high is 1 m for 16.75 river km, for P85high maximum water level attenuation is 0.26 m over the same distance). The gradients of the trend lines (i.e. the rates of attenuation shown by the dotted lines in Fig. 6b) are dependent on and follow a linear correlation with *H*_*max*_ values at the boundary condition (R^2^ = 0.96). These patterns may provide some predictive capability for maximum water level attenuation for comparable locations.

#### Maximum Wave Height

Maximum wave height, *H*_*max*_, differs from maximum water level, as it is a measure that extends from the peak to the trough of the largest wave in the water level time series at a particular point. This parameter is obtained from analysis of the tsunami time series at a given location and provides an indication of the depth range that would occur across a relatively short period of time.

**Figure 7:**
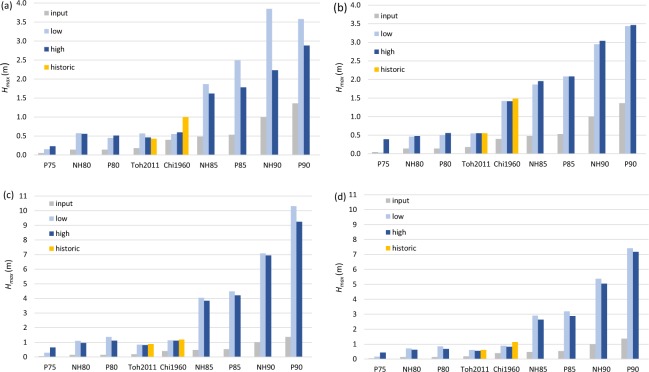
Maximum wave heights (*H*_*max*_) at the four case study locations for all modelled scenarios. (**a**) Spit Bridge (MSL depth = 6.1 m), (**b**) Anzac Bridge (MSL depth = 5.8 m), (**c**) Manly West (MSL depth = 9.2 m) and (**d**) Middle Harbour Entrance (MSL depth = 11.0 m). Note the scales on the y-axes differ between panels. The model boundary condition (i.e., the maximum wave height at the 100 m depth contour) for each scenario is shown by the ‘input’ column. See Fig. 1 for case study locations within the model domain. The legend specifies if the scenarios were modelled as coincident with low, high or historic tides. Historic tides represent the tide data from the time that a historic tsunami event occurred.

Manly West and Middle Harbour Entrance, the two most exposed of the examined locations (see Fig. 1), show the largest *H*_*max*_ values of the four example locations (Fig. 7c,d). The scenario with the largest maximum wave heights at the four case study locations is P90low, which results in *H*_*max*_ = 10.3 m at Manly West and 7.4 m at Middle Harbour Entrance. Scenarios P75, NH80, P80, Toh2011 and Chi1960 all have *H*_*max*_ < 1.5 m for all examined locations and for all tides modelled as shown in Fig. 7. The remaining scenarios NH85, P85 and NH90 show 1.6 < *H*_*max*_ < 7.0 m when including both low and high tide models.

Wave height amplification (relative to the wave height measured at the 100 m depth contour at the model boundary) is affected by tide-tsunami phasing and the degree of exposure within the estuary. The difference in wave height amplification between low and high tide models is greatest at the Spit Bridge location for scenarios NH85, P85, NH90 and P90 (Fig. 7). For example, for scenario NH90, *H*_*max*_ is amplified from 1 m at the model boundary to 2.2 m in the high tide model and to 3.9 m in the low tide model. The difference in maximum wave height between low and high tide models in all other locations is relatively minor for these scenarios, with differences between *H*_*max*_ at low and high tide for NH90 ranging between 0.1–0.3 m. Historic events with historic tides show amplification similar to or slightly greater than both low and high tide models, except for the Spit Bridge location, where the *H*_*max*_ value is amplified from 0.4 m to 1.0 m with historic tides, only 0.55 m with a low tide, and 0.59 m with a high tide.

### Model Uncertainty

**Figure 8:**
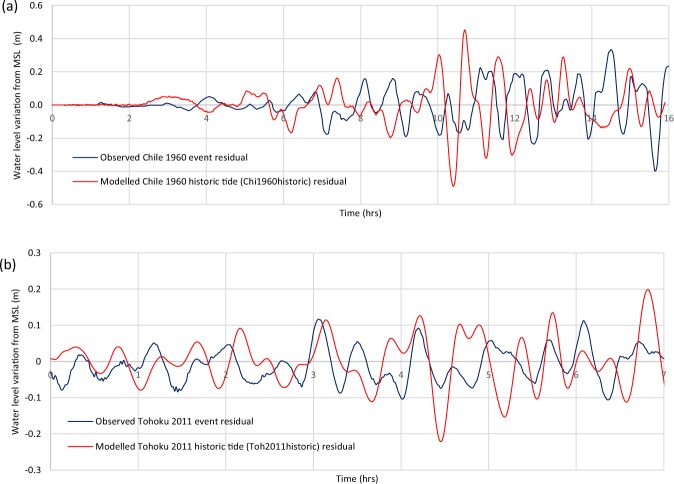
Water level time series comparing observed historical event residual data with modelled representations of historic events at the Fort Denison Tide Gauge: (**a**) Chile 1960 event time series (**b**) Tohoku 2011 event time series. Time ‘0’ represents tsunami arrival at the 100 m depth contour. See Fig. 1 for the Fort Denison Tide Gauge location.

To enable a degree of model validation, modelled results for both the 1960 Chile event and the 2011 Tohoku event were compared to observed residual data collected at the Fort Denison Tide Gauge (Fig. 8, Table 2). For both cases, the modelled results were found to overestimate wave heights. The sources of uncertainty are for the entire modelling process, from the rupture and deep-water propagation modelling (as extracted from T2), to the nearshore inundation modelling completed in this study. Thus, there is a wide range of potential error sources.

**Table 2:** Wave train parameters for both observed historical events and modelled representations of historical events.

Scenario	*H*_*rms*_ (m)	*H*_*max*_ (m)	*H*_*std*_ (m)
Observed Chile 1960 event residual	0.23	0.78	0.14
Modelled Chile 1960 (Chi1960) no tide	0.31	0.93	0.20
Modelled Chile 1960 historic tide (Chi1960historic) residual	0.28	0.94	0.24
Observed Tohoku 2011 event residual	0.12	0.20	0.06
Modelled Tohoku 2011 (Toh2011) no tide	0.22	0.24	0.04
Modelled Tohoku 2011 historic tide (Toh2011historic) residual	0.26	0.32	0.06

Wave height is measured from peak to trough and wave train parameters are defined as: root mean squared wave heights (*H*_*rms*_), maximum wave height (*H*_*max*_) and standard deviation of all wave heights (H_std_).

The observed *H*_*max*_ (*H*_*max*,*obs*_) at the Fort Denison tide gauge during the Tohoku 2011 event was 0.2 m and the modelled *H*_*max*_ (*H*_*max*,*mod*_) of the same event with no tide was 0.24 m, a difference of 0.04 m (*ΔH*_*max*_/*H*_*max*,*obs*_ = 20%). Modelling the same wave train with an historic tide increased *H*_*max*,*mod*_ to 0.32 m, increasing *ΔH*_*max*_ to 0.12 m (Δ*H*_*max*_/*H*_*max*,*obs*_ = 60%) (Fig. 8, Table 2). Both of these *ΔH*_*max*_/*H*_*max*,*obs*_ values for Tohoku 2011 at the Fort Denison tide gauge are within the range of *ΔH*_*max*_/*H*_*max*,*obs*_ values observed by Allen & Greenslade^35^ with their multiple comparisons of observed DART data to the deep water modelled representation of Tohoku 2011 used in this study (0.40 ≤ *H*_*max*,*obs*_ ≤ 1.13 m and 0.09 ≤ *ΔH*_*max*_ ≤ 0.22 m or 8% ≤ *ΔH*_*max*_/*H*_*max*,*obs*_ ≤ 38%)^35^, suggesting that the model used in this study does not increase overall uncertainty to a significant degree.

The modelled representation of the Chile 1960 event used in this study was not scaled to observed data but rather deduced from source data and some historic observations^36^. At the Fort Denison tide gauge *H*_*max*,*obs*_ = 0.78 m, *H*_*max*,*mod*_ = 0.93 m without tide and *H*_*max*,*mod*_ = 0.94 m with an historic tide (Fig. 8). The model therefore overestimated the maximum wave height by 0.16 m or ~20%. The *H*_*max*_ values for the models with and without tides for the Tohoku 2011 event differ by 0.08 m, whereas the *H*_*max*_ values for the models with and without tides for the Chile 1960 event differ by 0.01 m. It should be noted that incorporating tides into these models may not have a linear effect on the residual tsunami maximum wave heights.

## Discussion

This paper presents the most detailed assessment of the potential for earthquake-generated tsunami impact on Sydney Harbour to date. Such site specific modelling provides both scientists and emergency services with the most accurate estimation of tsunami impact possible. Results presented here can be used as an evidence base for both emergency planning and further research.

The tsunami threat to Sydney Harbour (Fig. 1) is primarily a marine one, with model results for all potential scenarios of 8.5 M_W_ and greater showing high current speeds, shallow water amplification of wave heights relative to deep water wave heights, and rapid changes in water depth; all of which are potential hazards for shipping and other water users. Of the potential tsunami modelled, land inundation is only an extensive threat for tsunami generated by earthquakes of 9.0 M_W_ (Fig. 2); although at Manly, minor inundation does occur for tsunami generated by earthquakes of 8.5 M_W_ and greater. The vulnerability of Manly to inundation shown here (Fig. 3) corresponds to observations from previous research^14,29^. The degree of land inundation is shown to be tide dependent, thus the time of year (spring or neap tides) as well as tide-tsunami phasing will directly affect the inundation hazard. For Sydney Harbour, inundation tends to primarily occur along the south side of the Harbour, which has relatively low-lying and gently sloped elevation; very little inundation occurs along the north side, where there is a more steeply sloped shoreline (Fig. 2).

Maximum area of inundation across the entire model domain correlates very closely (R^2^ = 0.99) with *H*_*max*_ and other wave height parameters (e.g. for H_*rms*_, R^2^ ≥ 0.94) (Fig. 2c) indicating that wave height parameters at the 100 m depth contour can provide reliable predictive data for this location. The non-linear polynomial relationships could also be used to expand on minimal data that exists for other analogous locations. The 80^th^ percentile runup values show a weak linear relationship with wave height parameters at the 100 m depth contour (R^2^ < 0.3). The gradients or attenuation rates for each scenario however, show a linear correlation to wave height parameters at the 100 depth contour (e.g. for *H*_*max*_, R^2^ = 0.95), indicating that overall runup attenuation rates may be predicted well, however runup values are more erratic. Runup attenuation occurs at a significantly slower rate (e.g. for the 80^th^ percentile runup values, 1 m attenuation over 5.5 km) than previous attenuation rules developed by Leonard *et al*.^30^ in Samoa (1 m attenuation over 0.4 km up-river). Such data suggests that runup attenuation may be very site-specific and dependent on geomorphology. Runup attenuation patterns shown in this study may only be comparable for other drowned river valleys of similar dimensions.

Within the geography of the Harbour, high maximum current speeds generated by tsunami predominantly occur in locations exposed to the open ocean. However, the geomorphology of a location appears to have a greater influence on where the highest maximum current speeds occur than the degree of exposure to the open ocean (Fig. 4). For the ≥8.5 M_W_ potential scenarios, maximum current speeds exceeded 4 m/s (as is considered destructive by NOAA^37^ at both the Spit and Anzac Bridges, which are locations that are somewhat protected from the tsunami but are characterised by narrow, shallow channels (Fig. 4). For the less protected and deeper locations, Middle Harbour Entrance and Manly West, maximum current speeds only exceeded 2 m/s for the low tide models (considered dangerous by NOAA^37^). Although the relative maximum current speeds are almost double in the narrow channels when compared to exposed but deeper locations, when combined with rapid changes in water level and wave height increases, the potential for damage is high across all of the examined locations. For the ≤8.0 M_W_ potential scenarios, maximum current speeds are shown to be hazardous only in a few, specific locations. For example, at the Spit Bridge, results show maximum current speeds exceed 2 m/s for all scenarios except P75low.

Bystander estimates of maximum current speeds during the 1960 Chilean historic event greatly exceed all the Chi1960 results obtained by this study. Historic accounts of the 1960 Chilean event included visual estimates of current speeds of 5–15 m/s at the Spit Bridge and 4–6 m/s at Anzac Bridge^15^. Model results for Chi1960low, high, and historic scenarios resulted in maximum current speeds of 3.1 m/s, 2.9 m/s, and 3.1 m/s respectively at the Spit Bridge and 2.6 m/s, 1.6 m/s, and 2.6 m/s at the Anzac Bridge (Fig. 4). The discrepancy between bystander observations and model results may be due to inaccurate visual estimates or inaccuracies within the modelled scenarios; however, the comparison of modelled and observed data, which shows that the model is slightly prone to overestimation of wave heights suggests that bystander overestimation of current speeds is likely.

Amplification of deep water wave heights is greatest at the most exposed locations within the estuary. Maximum water levels are consistently highest at the most exposed bays, where there is a shallow, funnelling channel that does not allow the through flow of water (Fig. 6a). Maximum water level attenuation along the two thalwegs drawn in Fig. 6c show a sensitivity to narrowing geomorphology or bottlenecks, such as that shown by the Spit Bridge along the northern thalweg (Fig. 6a,c). In the absence of significant narrowing, maximum water level attenuation follows a linear trend, with attenuation rates for each modelled scenario correlating well with 100 m depth contour wave height parameters. Maximum water level attenuation rates are, again, significantly slower (e.g. P90high maximum water levels attenuate at a rate of 1 m per 16.75 km, P85high maximum water levels attenuate at a rate of 1 m per 64 km) than rates shown by Leonard *et al*.^30^ in Samoa (maximum water levels attenuate at a rate of 1 m per 0.4 km). Similar to runup attenuation rates, site specificity appears to be significant and detailed attention must be paid to geomorphology when considering transferring attenuation rates between sites.

Of the locations examined for maximum wave height (*H*_*max*_), the largest value exceeds 10 m at Manly West (P90low) (Fig. 7), over seven times the maximum wave height of the boundary condition. The two examined locations that are relatively protected, Spit Bridge and Anzac Bridge, show a lesser degree of wave height amplification. For the same scenario described above, P90low, both these locations experience maximum wave heights under 4 m (less than three times the boundary condition maximum wave height), indicating significant wave height attenuation occurs after the initial shoaling at the Harbour entrance.

Historic records provide an opportunity for a numerical comparison with model results to assist with quantifying the uncertainty of the model. The comparison between the observed and modelled results for the Chile 1960 and Tohoku 2011 events at the location of the Sydney Fort Denison Tide Gauge show that the model overestimates wave heights for these events (Fig. 8). The comparison between observed and modelled residual data for the Tohoku 2011 event shows Δ*H*_*max*_ values (without tide: *ΔH*_*max*_ = 0.04 m and *ΔH*_*max*_/*H*_*max*,*obs*_ = 20%; with tide: *ΔH*_*max*_ = 0.12 m and *ΔH*_*max*_/*H*_*max*,*ob*_ = 60% of *H*_*max*,*obs*_) similar to the range of *ΔH*_*max*_ values calculated from the modelled representation of this event and the observed DART data used for scaling (0.09 ≤ Δ*H*_*max*_ ≤ 0.22 m; 8% ≤ Δ*H*_*max*_/*H*_*max*,*obs*_ ≤ 38%)^32^. This suggests that uncertainty has not significantly increased between the DART locations and the Fort Denison tide gauge location (*i.e*. within the modelling presented here).

The Tohoku 2011 event modelled was scaled to several DART observations^35^, which was not possible with the Chile 1960 event^36^. The comparisons of observed data to the Tohoku 2011 and Chile 1960 modelled events are therefore not equivalent and the Tohoku 2011 comparison may be the more reliable indication of model uncertainty at the Fort Denison tide gauge. The overestimation of the Tohoku 2011 modelled event appears to be consistent across the rest of the Sydney Harbour model in this study. Model results showed impact parameters closer in severity to those of Chile 1960 than what happened historically (no dangerous conditions were reported in Sydney Harbour during the historic 2011 Tohoku event).

The modelled representation of Chile 1960 also overestimated wave heights when compared to historic observations (*ΔH*_*max*_ = 0.16 m; Δ*H*_*max*_/*H*_max,obs_ = 20%). Comparisons of T2 modelled data and observed DART data for the examples described in Greenslade *et al*.^38^ provide some indication of the T2 overall model uncertainty, although not specifically for the Chile source. The seven comparisons between observed DART data and modelled T2 data show a mean *ΔH*_*max*_/*H*_max,obs_ of 20%, although this comparison should consider the notably small waves (*H*_*max*_ < 0.07 m; 0 ≤ Δ*H*_*max*_ ≤ 0.02 m)^38^. From the data available, it appears that model uncertainty for this study has not significantly increased from the T2 database output to the Fort Denison tide gauge.

The work of Allen & Greenslade^35^ at nearby Port Kembla (~75 km south of Sydney Harbour) shows model uncertainty for both the T2 deep ocean modelling and their additional nearshore modelling and so provides an overall deep ocean and nearshore modelling uncertainty comparison. The tsunami wave trains in Allen & Greenslade^32^ were modelled without tides. *ΔH*_*max*_ at the more reliable outer harbour location for the Chile source event (Chile 2010) was 0.43 m (*H*_*max*,*obs*_ = 0.58 m; *H*_*max*,*mod*_ = 1.01 m; *ΔH*_*max*_/*H*_*max*,*obs*_ = 73%) and for the Tohoku 2011 event (named Honshu 2011 in the study) was 0.23 m (*H*_*max*,*obs*_ = 0.6 m, *H*_*max*,*mod*_ = 0.83 m, *ΔH*_*max*_/*H*_*max*,*obs*_ = 38%)^35,39^. When compared to the two historic models without tide in this study (both Chile 1960 and Tohoku 2011 *ΔH*_*max*_/*H*_*max*,*obs*_ = 20%), the data from Allen & Greenslade^38^ shows a greater model overestimation of maximum wave heights.

The use of these quantitative assessments comparing observed and modelled data must take into account the different spatial scales of variability, issues may therefore have also arisen from small phase errors, which could lead to the comparison of less suitable grid points for the observations^38^.

Earthquake source parameters have been shown to be important for determining uncertainty in tsunami hazard. Assuming a uniform slip on the rupture interface in tsunami models, as was done for this study, can underestimate the potential impact and extent of inundation by an equivalent magnitude of about 0.3 or 0.4^40,41^. It has been shown that simulation of an 8.7 M_W_ earthquake with uniform slip reproduced the area that could be inundated by equivalent non uniform slip events of 8.4M_W_^41^. It follows that similar underestimations could have occurred in this study. Beyond slip uniformity, relying on one particular inversion model as a representative case has also been shown to underestimate the total tsunami hazard and potential consequences^42^. This variability is caused by variations in the initial ocean surface displacement, which when using MOST^43–45^ as we do, is assumed to be equal to the displacement of the seafloor. For a given rupture depth and dip, heterogeneous slip ocean surface displacements have greater amplitude and steepness, which can lead to greater tsunami inundation^41,46^. The volume and range of ocean displacement has also been shown to correlate with tsunami runup height^47^.

The elevation datasets created for this study are derived from the best data available. However, instrumentation used to collect this data contains inherent uncertainty as does the processing required to produce gridded datasets^48^. This uncertainty increases with depth^49^ but the influence of the bathymetry on the tsunami wave is greater in shallower water^8^. Compilation datasets such as the nearshore dataset created for this study are derived from numerous instruments, processing origins and with varying degrees of metadata. As a result, the shallow water dataset compiled for this study is unlikely to meet the criteria for an IHO Order 2 survey, which requires a maximum total vertical uncertainty of 1 m, with a 95% certainty of 0.023m^49^.

Spatially variable friction^50^ and the permeability of buildings^51^ are known to affect tsunami impact prediction including runup and inundation extent patterns. Experimentation by Cardno^29^ provides some confidence in the friction applied to this model domain, however, there is likely to be some overestimation of inundation extent for areas with buildings for the broad scale model. For the Manly model, which uses very high resolution topography that is able to resolve building structure, building permeability is likely to be the greater source of uncertainty.

Further limitations within this study include the positional data used to extract model results representing the Fort Denison Tide Gauge, which were extracted ~90 m away from the true position of the instrument. This was due to the tide gauge being enclosed and within the island domain of Fort Denison, which is above MSL in the model due to the spatial resolution of the model. Another limitation to this study and tsunami modelling as a whole is that all widely used hydrodynamic models employ a static elevation model, which does not account for sediment transport. Significant scouring was reported during the 1960 Chile event, including that which removed a large section of beach near the Spit Bridge^15^. Such large scale erosion would likely influence the parameters detailed in our results, however, concurrent sediment modelling was not possible to incorporate into the modelling software.

From the detailed analysis of potential tsunami impact to Sydney Harbour presented here, we conclude that for locations with similarly distanced source zones and analogous geomorphology, earthquakes of 8.5 M_W_ and greater pose the most impactful threat, including to land at low lying elevations. Tsunami sourced from earthquakes of ≤8.0 M_W_ are likely to cause a threat almost exclusively to marine traffic and infrastructure. We also show that inundation and maximum depth levels are a greater threat if peak tsunami waves occur at high tide. Should peak tsunami waves coincide with low tide, minimum depth levels are of concern, particularly for shallow navigation channels with limited under-keel clearance. Within the estuary, the geomorphology is shown to be a greater influence on the location of the highest maximum current speeds than the degree of exposure to the open ocean, with narrow, shallow channels or bottlenecks in the Harbour showing the greatest vulnerability even up to 11 km upstream. These bottle necks also cause elevated water levels. Maximum current speeds are more likely to be a threat for tsunami coincident with low tide but are potentially a threat for all events. Wave height increase is greatest for exposed locations but continues to be a hazard upstream as far as 11 km from the estuary mouth.

Maximum tsunami wave heights at the 100 m depth contour, which are available in the T2 database for numerous scenarios Australia wide, can be used to predict severity of impacts. Specific correlations include a second order polynomial relationship between *H*_*max*_ at the 100 m depth contour and inundation area as well as linear relationships between *H*_*max*_ at the 100 m depth contour and up-river attenuation rates for runup and maximum water levels.

These results and subsequent conclusions greatly contribute to the evidence base available for tsunami preparedness in NSW and, as such, are of significance to Australia’s efforts to understand this hazard. Attenuation rules and patterns provide some insight into tsunami behaviour in drowned river valley estuaries that may be applicable elsewhere. Our conclusions therefore provide further knowledge of the potential for tsunami impact in other estuaries with similar morphology and similarly distanced source zones.

## Methods

The inputs required for the hydrodynamic modelling used in this study include bathymetry/topography, tsunami wave train boundary conditions, tide data and friction values. Details for these specific inputs are provided in this section. ANUGA was selected as an open source hydrodynamic model suitable for these tsunami inundation models. ANUGA uses the DE1 flow algorithm^52^ and has been validated and used extensively to model the hydrodynamics of tsunami^53–55^. ANUGA solves the non-linear shallow water wave equations, which have been shown to best represent tsunami behaviour^44^.

### Bathymetry and Topography Data

Bathymetry and topography data were collected from numerous sources and in resolutions from 1–250 m. The software ESRI ArcGIS 10.1™ was used to shift or project the source data so that all data were collated at Australian Height Datum (AHD) and WGS84 UTM zone 56. The data were then gridded at 10 m, which was the highest resolution possible without eroding accuracy. For areas with data gaps, interpolation was required, using the ESRI tool, Topo to Raster. A data descriptor of this dataset with associated metadata has been published with DEM data downloadable from Pangaea^56^.

Resolutions within the model were selected by convergence testing for maximum accuracy of results whilst conserving computing load. Within the primary area of interest, the triangular mesh generated by ANUGA had triangles with a maximum area of 450 m^2^. The Manly sub model generated a triangular mesh with a maximum triangle area of 2 m^2^.

### Tsunami Wave Train Boundary Conditions

Wave train boundary conditions were sourced from the T2 tsunami scenario database^27^, which was developed by the JATWC in order to facilitate nearshore tsunami modelling and improve tsunami warning capabilities. The database provides over 1,800 tsunami scenarios from global sources.

For the generation of the T2 database, rupture modelling uses a uniform slip rupture and is based on the relationship between magnitude and rupture dimensions^27^. The MOST model developed by Titov and Synolakis^44^ is used to determine seafloor deformation and therefore the initial condition for tsunami propagation. MOST assumes a double-couple source model, in which the earthquake is assumed to consist of two orthogonal shears with opposite sign^43,45^. For the initial condition, the initial sea surface elevation is identical to vertical seafloor displacement and the ruptures are assumed to occur instantaneously. Horizontal seafloor displacements were not used to calculate the initial conditions^39,44^.

The details of the tsunami wave train earthquake source zones modelled are provided in Tables 3 and 4. Table 3 provides the locations of the source zones and Table 4 provides the details of the initial conditions. Initial conditions were derived from the relationship between magnitude and rupture dimensions as described below, where magnitude (*M*_*W*_) is related to seismic moment (*M*_*o*_) as:

1 $${M}_{W}=\frac{2}{3}(lo{g}_{10}{M}_{o}-9.1)$$

and seismic moment is related to the rupture characteristics of the earthquake as:

2 $${M}_{o}=\mu LW{u}_{o}$$

where *µ* is the shear modulus and *L*, *W* and *u*_*o*_ are the length, width and slip of the rupture respectively (in metres). In equation 2, *µ* is taken to be 4.5 × 10^10^ Nm^−2^. The rupture elements are not identical for each magnitude event as the widths and slip of the ruptures are different for each magnitude^27^.

**Table 3:** Tsunami Source Zone locations. Since initial conditions cover a large area, positions are provided as the mean latitude and longitude of the two central rupture elements used to determine initial conditions.

Source Zone	Latitude	Longitude	Source Reference	Scaling Factor
New Hebrides	20°08′S	168°46′E	Bird^62^	none
Puysegur	47°01′S	165°52′E	Bird^62^	none
Chile 1960	41°45′S	73°43′W	Bird^62^; Plafker^66^	2.00
Tohoku 2011	38°51′N	142°52′E	Bird^62^	5.62

**Table 4:** Details of the initial conditions used for the T2 scenario database.

Magnitude (M_W_)	Seismic moment (*M*_*o*_) (Nm)	Width (*W*) (km)	Length (approximate) (*L*) (km)	Slip (*u*_*o*_) (m)	Source Reference
**T2 Scenarios**					
7.5	2.24 × 10^20^	50	100	1	Greenslade *et al*.^27^
8.0	1.26 × 10^21^	65	200	2.2	Greenslade *et al*.^27^
8.5	7.2 × 10^21^	80	400	5	Greenslade *et al*.^27^
9.0 (New Hebrides)	4.0 × 10^22^	100	1000	8.8	Greenslade *et al*.^27^
9.0 (Puysegur)	3.97 × 10^22^	100	600	14.7	Simanjutak *et al*.^59^
**Historic Events**					
Chile 1960 (9.0)	7.92 × 10^22^	100	1000	17.6	Greenslade (*pers*. *comm*.)^39^
Tohoku 2011 (9.0)	4.05 × 10^22^	80	400	28.1	Greenslade (*pers*. *comm*.)^39^

The two historic events modelled in this study were also generated by the MOST model with the same assumptions as the hypothetical New Hebrides and Puysegur events modelled. The Tohoku 2011 scaling was determined by deep water observations from DART^57^, specifically 21413, 21419, 21415, 21414 and 52402. The most suitable initial conditions (Table 4) were also chosen to best resemble the deep water observational in preference to best resembling the earthquake that took place^35^.

There were no deep water observations available for the Chile 1960 event. A scaling factor of 2 was used to scale the 9.0 M_W_ initial conditions detailed in Table 4 to 9.2 M_W_. Although the historic event is considered to have been a 9.5 M_W_ event^58^, scaling beyond 9.2 M_W_ is not considered suitable^27^. Scaling this event to 9.5 M_W_ (with a scaling factor of 5.6) was shown to overestimate impacts reported compared to those observed in Dominey-Howes^11^.

The scaling factor for the Chile 1960 event was derived by assuming that the only difference between the rupture of the pre-computed scenario (with magnitude *M*_1_) and the rupture of the new earthquake (with magnitude *M*_2_) was the slip^27^. The scaling factor, *F*_*s*_, can be derived from Equations 1 and 2 ^27^, as

3 $${F}_{s}={10}^{\frac{3}{2}({M}_{2}-{M}_{1})}$$

MOST is also used to model tsunami propagation from source to location^27^. The horizontal grid spacing for T2 is 4 arc minutes and due to the convergence of longitude lines, this means that the actual spatial resolution ranges from ~7 km at the equator to 2.6 km at 69.4°S. Through the Courant-Friedrichs-Lewy (CFL) criterion, this 2.6 km grid size imposes a limit of 12 seconds on the time step. Within the MOST model, tsunami energy propagates freely through open boundaries with no reflection (apart from some minor numerical effects in some cases). The Antarctic ice-edge is represented as a land boundary. Model run-time for each scenario is 24 hours to ensure that reflections off underwater features or distant coasts are captured^59^. Bottom friction terms are not included as they are considered to be negligible in deep water^25,60^.

For this study, tsunami time series were extracted from the MOST model at a location along the 100 m depth contour offshore Sydney Harbour. These time series were then used as a boundary condition (at the 100 m depth contour) for the ANUGA tsunami inundation models presented in this study (Fig. 1). Scenarios selected were from the two source zones known to be the greatest threat to Sydney Harbour: the Puysegur and New Hebrides trenches^33^. Although it is recognised that seismic moment may be more closely correlated to tsunami intensity, earthquake source magnitude was used to select tsunami wave trains for this study. Earthquake source magnitude is the parameter used as reference by the T2 database and the JATWC^27^. This study aims to contribute to Australia’s national warning system and so follows the same convention. The results therefore show the hazard variables with respect to earthquake magnitude. A range of magnitudes is assessed to encompass low to high impact events. Tsunami time series scenarios 7.5, 8.0, 8.5 and 9.0 M_W_ from the Puysegur source and 8.0, 8.5 and 9.0 M_W_ from the New Hebrides source were extracted from T2. The 7.5 M_W_ scenario from New Hebrides was considered too small to cause any impacts of consequence and was not included.

The range of magnitudes assessed here is supported by global studies^61^ with the possible exception of the Puysegur 9.0 M_W_ event. The Puysegur subduction zone, south of New Zealand at ~600 km long^62^ is relatively short in length and has generally been considered unable to support a 9.0 M_W_ earthquake, with maximum possible estimates of <8.5M_W_^63^. Recent large events however, have demonstrated that very large earthquakes can be generated with ruptures shorter than 1000 km. The Chile 2010 8.8 M_W_ event had a rupture length of ~400 km^64^ and the Japan (Tohoku) 2011 9.0 M_W_ event had a rupture length of ~300 km^65^. One of the consequences of the underestimation of this source zone was that numerous fatalities occurred outside the regions marked as major inundation zones in public tsunami hazard maps^42^. Global studies have also stated that in the context of recurrence times and our short history of observation, we cannot rule out a >9.0 M_W_ at any subduction zones^61^. In the interests of providing data to support the JATWC, which has considered it necessary to include the Puysegur 9.0 M_W_^59^ event in the national database, it was deemed appropriate to model a 9.0M_W_ event from the Puysegur subduction zone in this study.

### Tide Data

Tide data used were extracted from one tidal cycle, from the spring tides of January 2015 at the Middle Harbour Tide Gauge (151°15′30.72′′, −33°49′31.56′′). The tide cycle showing the largest wave height (*H*_*max*_) of those spring tides was selected. Tide data was converted to MSL from the local datum, Zero Fort Denison. T2 data and this tide data were added together to provide dynamic tide input boundary conditions for modelling. One model was completed with only the tide input as a boundary condition for comparison and high tide level indication. The data from this tide only model, at the Middle Harbour Tide Gauge location differed from the original tide data by 0–0.07 m, a mean of 0.01 m.

The duration of the T2 tsunami scenarios varied, however the most impactful waves occurred over a 6 hour time window. The tidal period in Sydney Harbour covering both a high and low tide is approximately 12 hours and so T2 scenarios were summed so that the largest waves were coincident with both a high tide and a low tide.

### Friction

For the broad scale models a Manning’s value of 0.02 was considered suitable for the model domain. Experimentation has shown this value to be applicable for seabed roughness, road surface and sand/gravel^29^. For the high resolution model focusing on Manly, high resolution LIDAR data was used to create a more complex elevation dataset that was detailed enough to represent buildings. This method has been shown to be an accurate way of modelling tsunami inundation in built up areas^53^. This is confirmed by inundation results for the Manly model showing inundation occurring along individual streets between mapped buildings.

## Electronic supplementary material

Supplementary Information

## Data Availability

The datasets generated and/or analysed during the current study are available from the corresponding author on reasonable request.
