# Supplementary material for: The Tsunami Threat to Sydney Harbour, Australia: Modelling potential and historic events
Source: Sci Rep. 2018 Oct 15;8:15045. doi: 10.1038/s41598-018-33156-w (PMC6189182; doi:10.1038/s41598-018-33156-w)

Supplementary Information

The Tsunami Threat to Sydney Harbour, Australia: Modelling potential and historic events.

Kaya M. Wilson[1]*, Stewart C. R. Allen[2], and Hannah E. Power[1]

[1]School of Environmental and Life Sciences, The University of Newcastle, Callaghan, Australia.

[2]Bureau of Meteorology, Melbourne, Australia.

*Corresponding authors; kaya.wilson@newcastle.edu.au, hannah.power@newcastle.edu.au

Supplementary Figure S1: Map of maximum inundation for event P90high. *Inundation is shown above the spring high tide line, which is derived from the tide only model. See Methods: Tide Data for information on the tide data used. This image was created by KMW using ESRI ArcMap 10.3.1 http://www.esri.com/arcgis/about-arcgis, coastline data (https://ecat.ga.gov.au/geonetwork/srv/eng/search#!a05f7892-eae3-7506-e044-00144fdd4fa6) from © Commonwealth of Australia (Geoscience Australia) 2017 and satellite imagery Landsat 8 couresy of the U.S. Geological Survey (https://earthexplorer.usgs.gov/).*

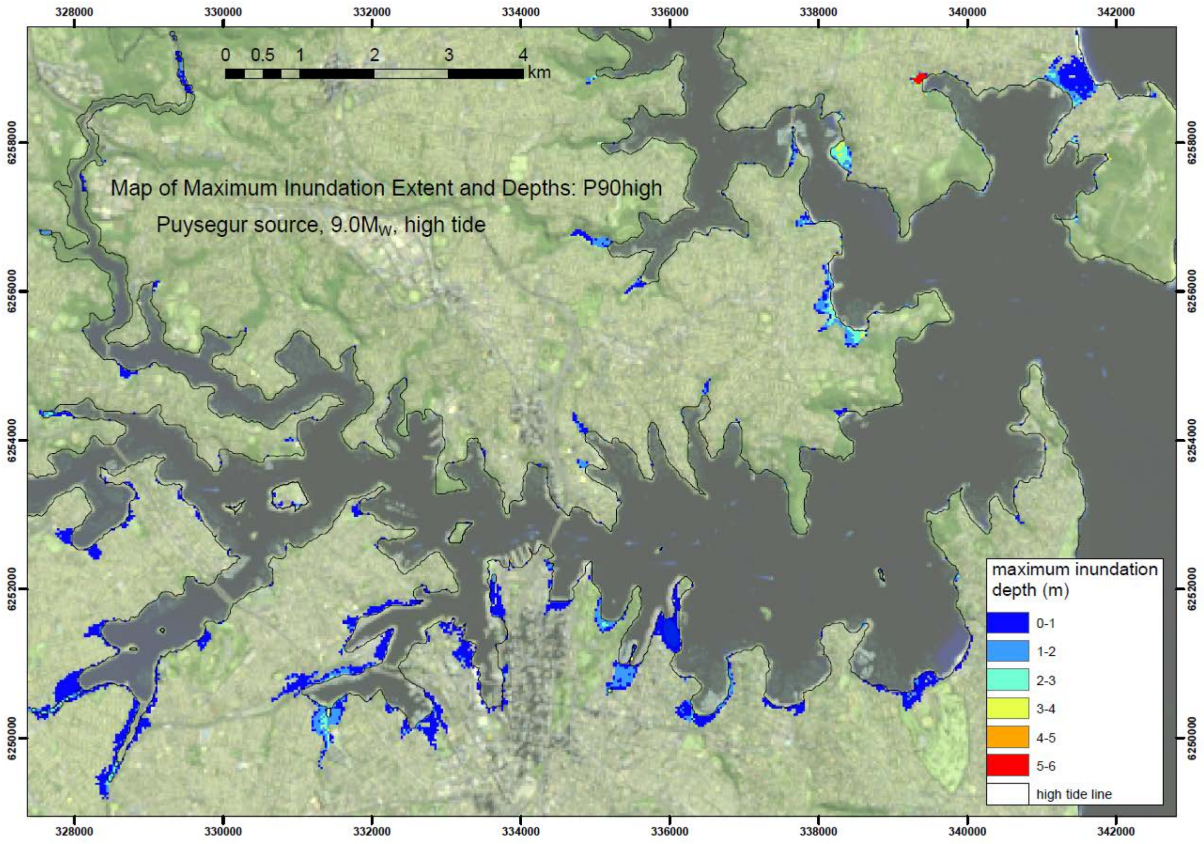

Supplementary Figure S2: Map of maximum inundation for event P90low. *Inundation is shown above the spring high tide line, which is derived from the tide only model. See Methods: Tide Data for information on the tide data used. This image was created by KMW using ESRI ArcMap 10.3.1 http://www.esri.com/arcgis/about-arcgis, coastline data (https://ecat.ga.gov.au/geonetwork/srv/eng/search#!a05f7892-eae3-7506-e044-00144fdd4fa6) from © Commonwealth of Australia (Geoscience Australia) 2017 and satellite imagery Landsat 8 couresy of the U.S. Geological Survey (https://earthexplorer.usgs.gov/).*

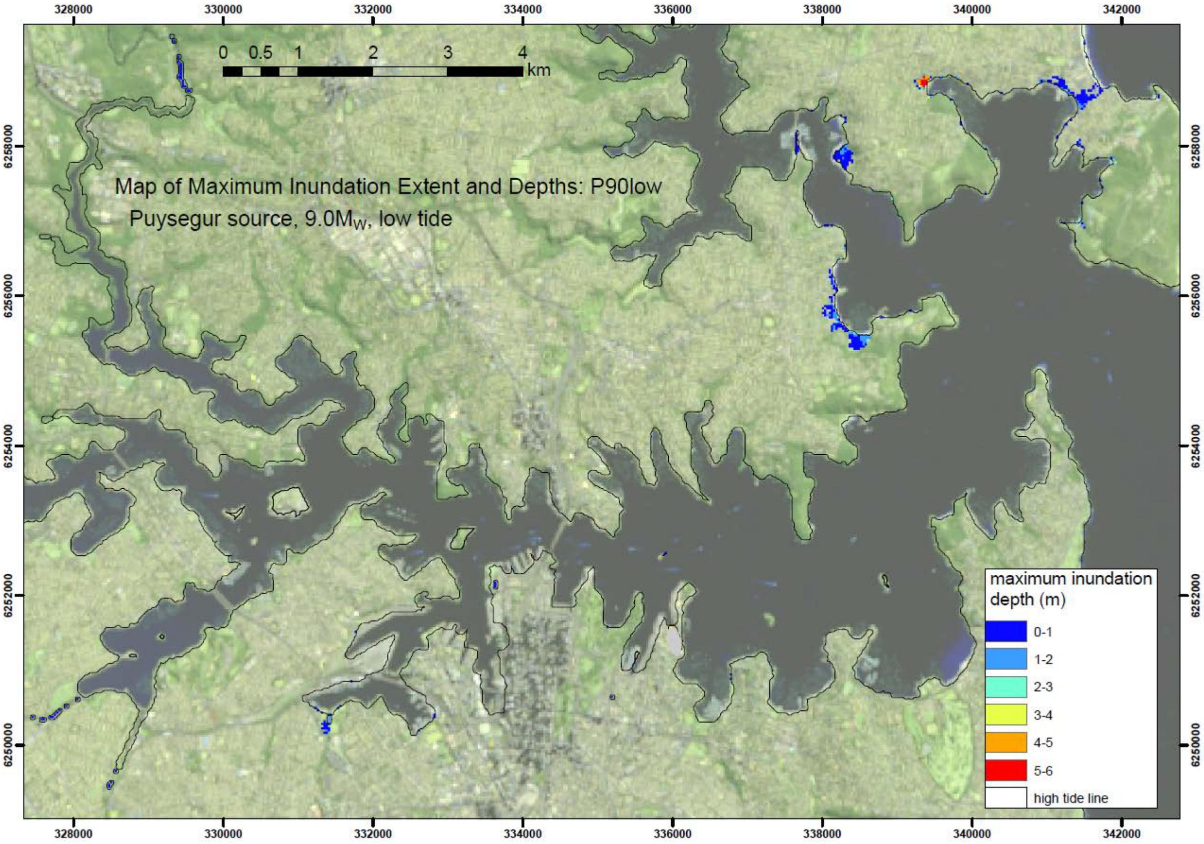

Supplementary Figure S3: Map of maximum inundation for event NH90high. *Inundation is shown above the spring high tide line, which is derived from the tide only model. See Methods: Tide Data for information on the tide data used. This image was created by KMW using ESRI ArcMap 10.3.1 http://www.esri.com/arcgis/about-arcgis, coastline data (https://ecat.ga.gov.au/geonetwork/srv/eng/search#!a05f7892-eae3-7506-e044-00144fdd4fa6) from © Commonwealth of Australia (Geoscience Australia) 2017 and satellite imagery Landsat 8 couresy of the U.S. Geological Survey (https://earthexplorer.usgs.gov/).*

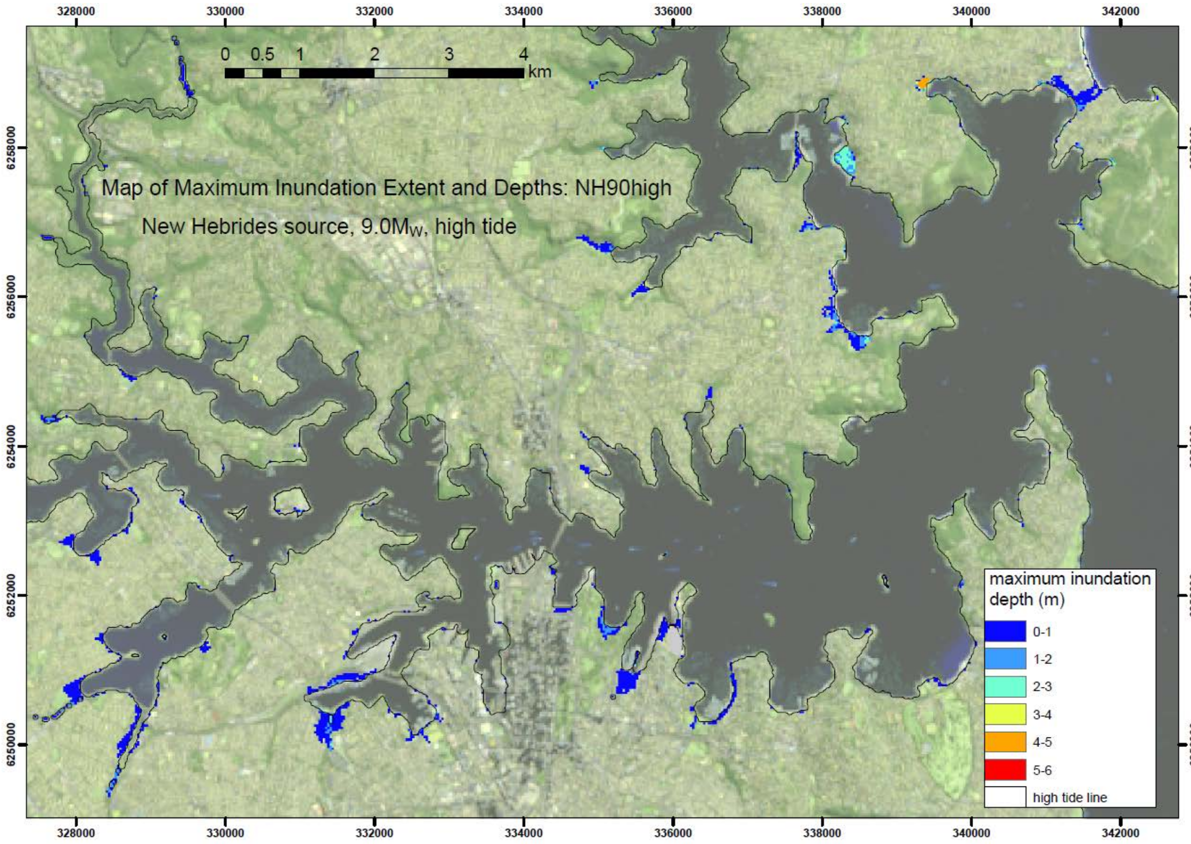

Supplementary Figure S4: Map of maximum inundation for event NH90low. *Inundation is shown above the spring high tide line, which is derived from the tide only model. See Methods: Tide Data for information on the tide data used. This image was created by KMW using ESRI ArcMap 10.3.1 http://www.esri.com/arcgis/about-arcgis, coastline data (https://ecat.ga.gov.au/geonetwork/srv/eng/search#!a05f7892-eae3-7506-e044-00144fdd4fa6) from © Commonwealth of Australia (Geoscience Australia) 2017 and satellite imagery Landsat 8 couresy of the U.S. Geological Survey (https://earthexplorer.usgs.gov/).*

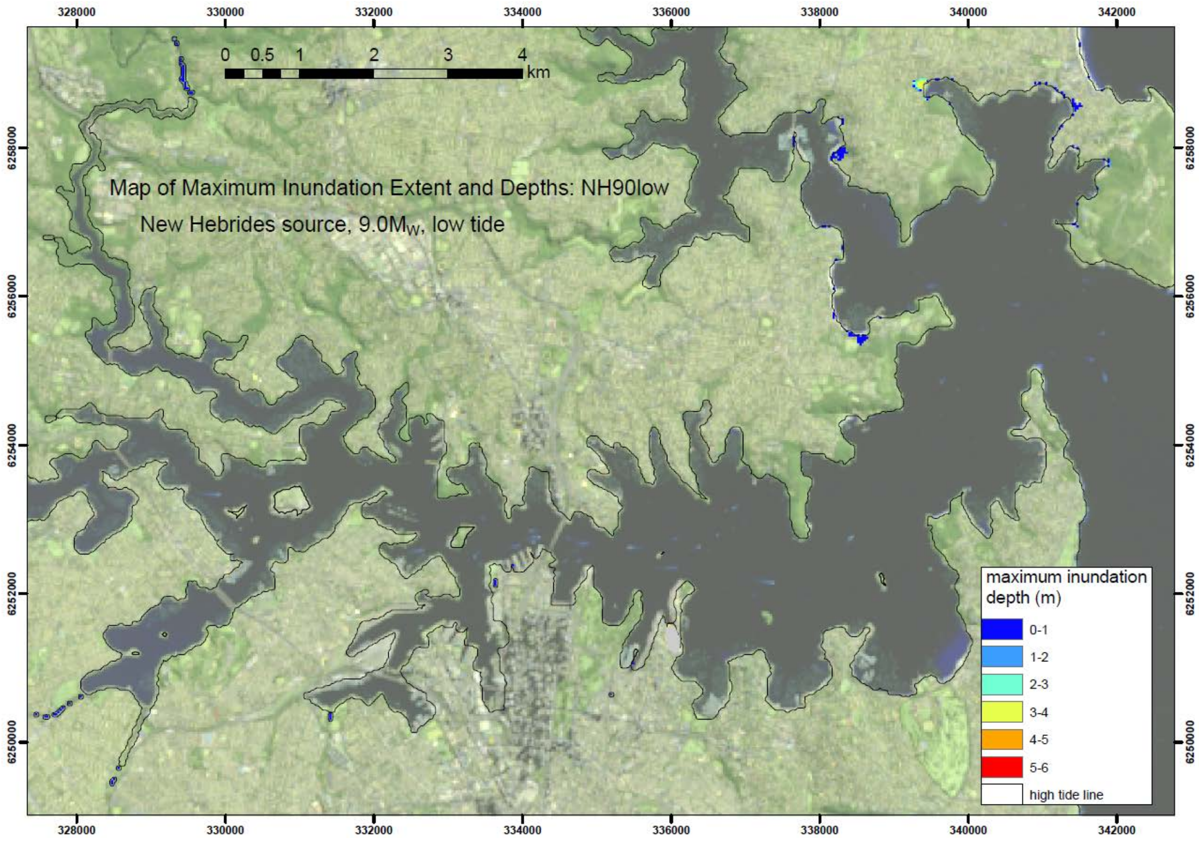

Supplementary Figure S5: Map of maximum inundation for event P85high. *Inundation is shown above the spring high tide line, which is derived from the tide only model. See Methods: Tide Data for information on the tide data used. This image was created by KMW using ESRI ArcMap 10.3.1 http://www.esri.com/arcgis/about-arcgis, coastline data (https://ecat.ga.gov.au/geonetwork/srv/eng/search#!a05f7892-eae3-7506-e044-00144fdd4fa6) from © Commonwealth of Australia (Geoscience Australia) 2017 and satellite imagery Landsat 8 couresy of the U.S. Geological Survey (https://earthexplorer.usgs.gov/).*

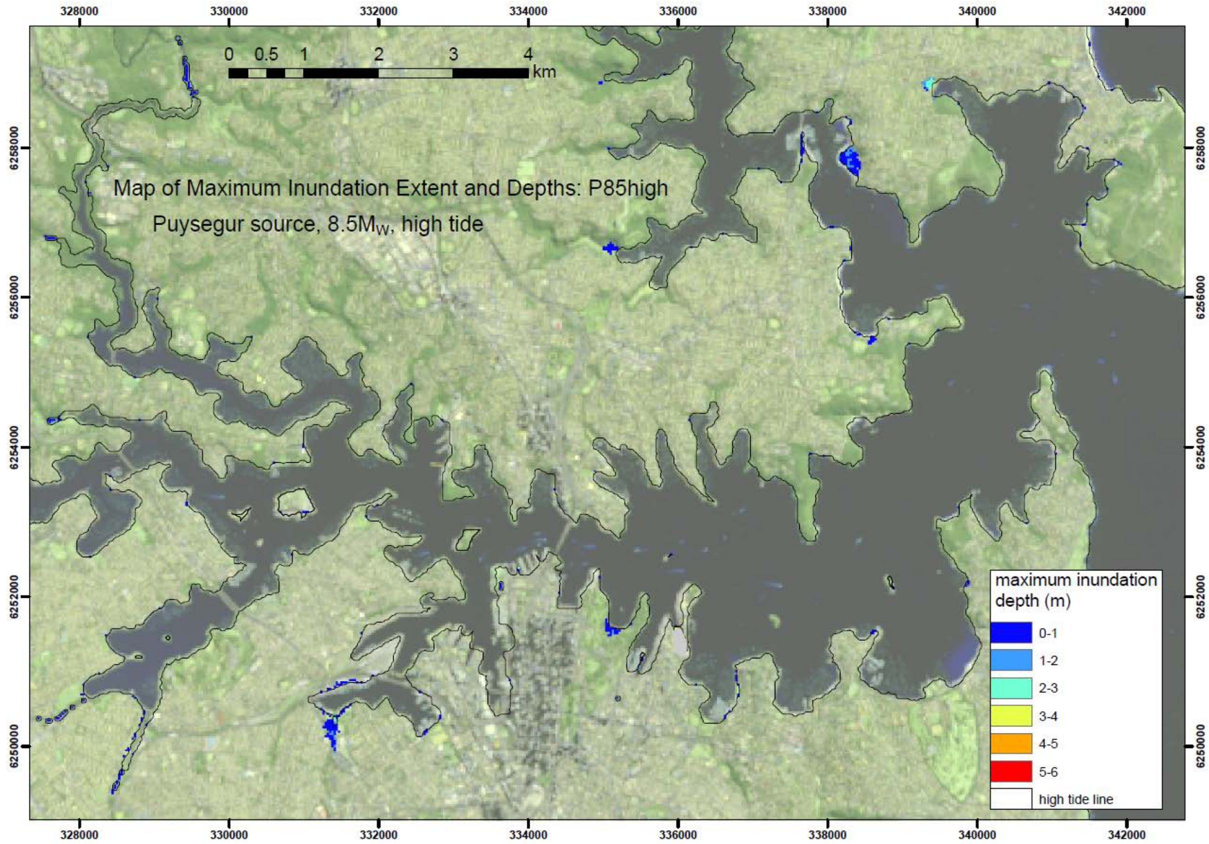

Supplementary Figure S6: Map of maximum inundation for event NH85high. *Inundation is shown above the spring high tide line, which is derived from the tide only model. See Methods: Tide Data for information on the tide data used. This image was created by KMW using ESRI ArcMap 10.3.1 http://www.esri.com/arcgis/about-arcgis, coastline data (https://ecat.ga.gov.au/geonetwork/srv/eng/search#!a05f7892-eae3-7506-e044-00144fdd4fa6) from © Commonwealth of Australia (Geoscience Australia) 2017 and satellite imagery Landsat 8 couresy of the U.S. Geological Survey (https://earthexplorer.usgs.gov/).*

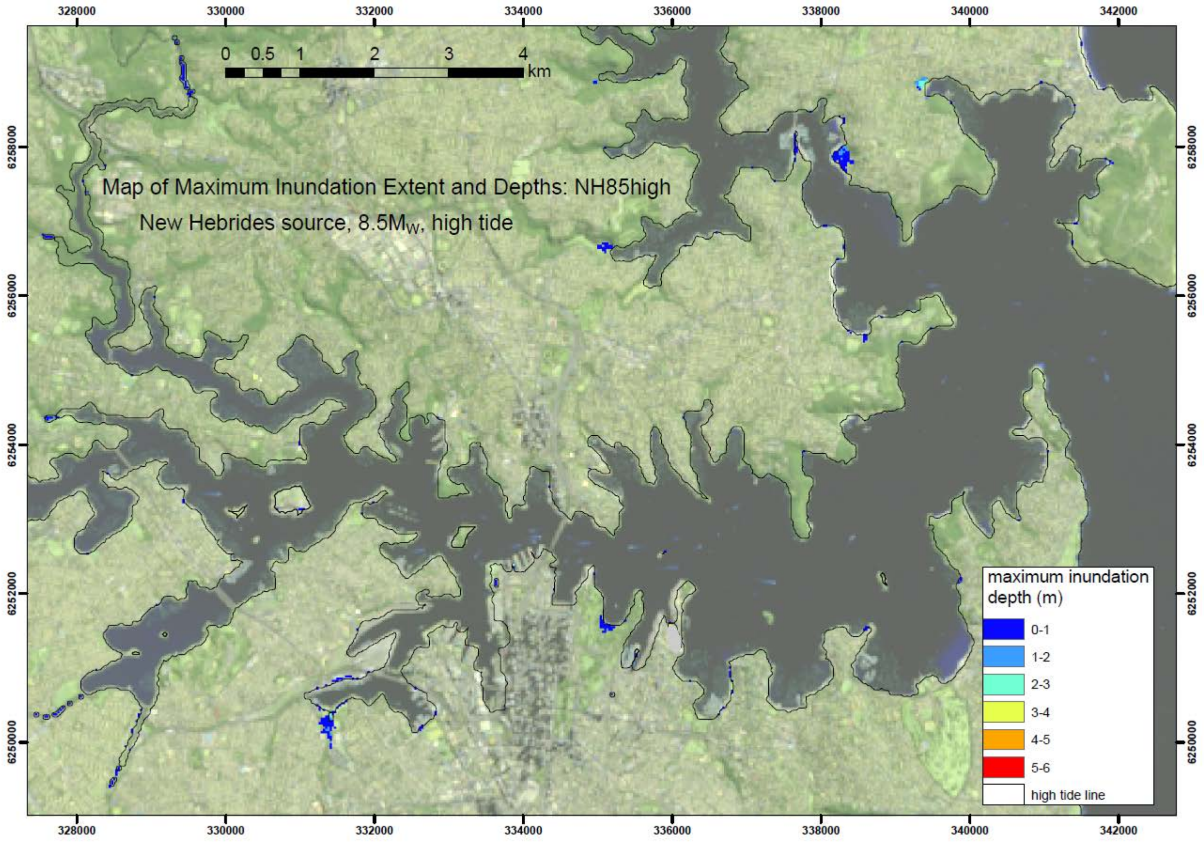

Supplementary Figure S7: Map of maximum inundation for event Chi1960high. *Inundation is shown above the spring high tide line, which is derived from the tide only model. See Methods: Tide Data for information on the tide data used. This image was created by KMW using ESRI ArcMap 10.3.1 http://www.esri.com/arcgis/about-arcgis, coastline data (https://ecat.ga.gov.au/geonetwork/srv/eng/search#!a05f7892-eae3-7506-e044-00144fdd4fa6) from © Commonwealth of Australia (Geoscience Australia) 2017 and satellite imagery Landsat 8 couresy of the U.S. Geological Survey (https://earthexplorer.usgs.gov/).*

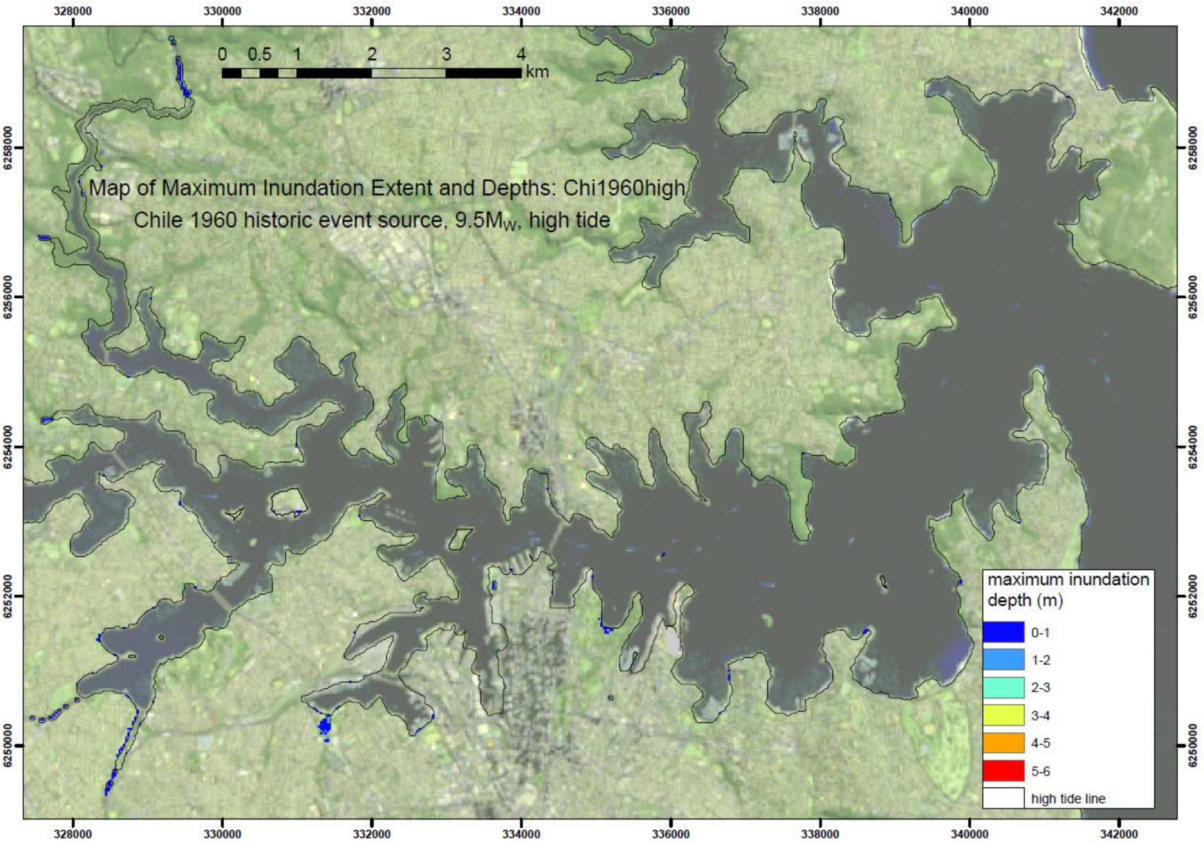

Supplementary Figure S8: Map of maximum current speeds for event P90high. *This image was created by KMW using ESRI ArcMap 10.3.1 http://www.esri.com/arcgis/about-arcgis, coastline data (https://ecat.ga.gov.au/geonetwork/srv/eng/search#!a05f7892-eae3-7506-e044-00144fdd4fa6) from © Commonwealth of Australia (Geoscience Australia) 2017 and satellite imagery Landsat 8 couresy of the U.S. Geological Survey (https://earthexplorer.usgs.gov/).*

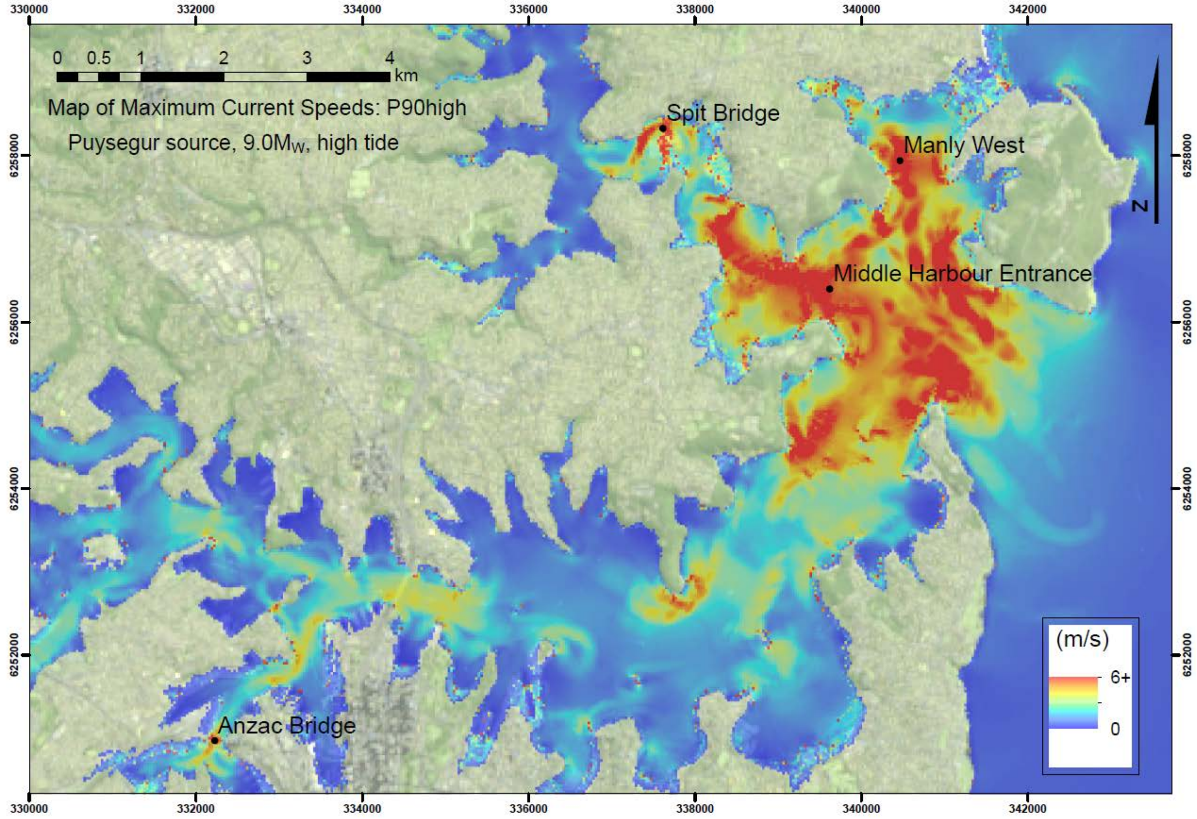

Supplementary Figure S9: Map of maximum current speeds for event P90low. *This image was created by KMW using ESRI ArcMap 10.3.1 http://www.esri.com/arcgis/about-arcgis, coastline data (https://ecat.ga.gov.au/geonetwork/srv/eng/search#!a05f7892-eae3-7506-e044-00144fdd4fa6) from © Commonwealth of Australia (Geoscience Australia) 2017 and satellite imagery Landsat 8 couresy of the U.S. Geological Survey (https://earthexplorer.usgs.gov/).*

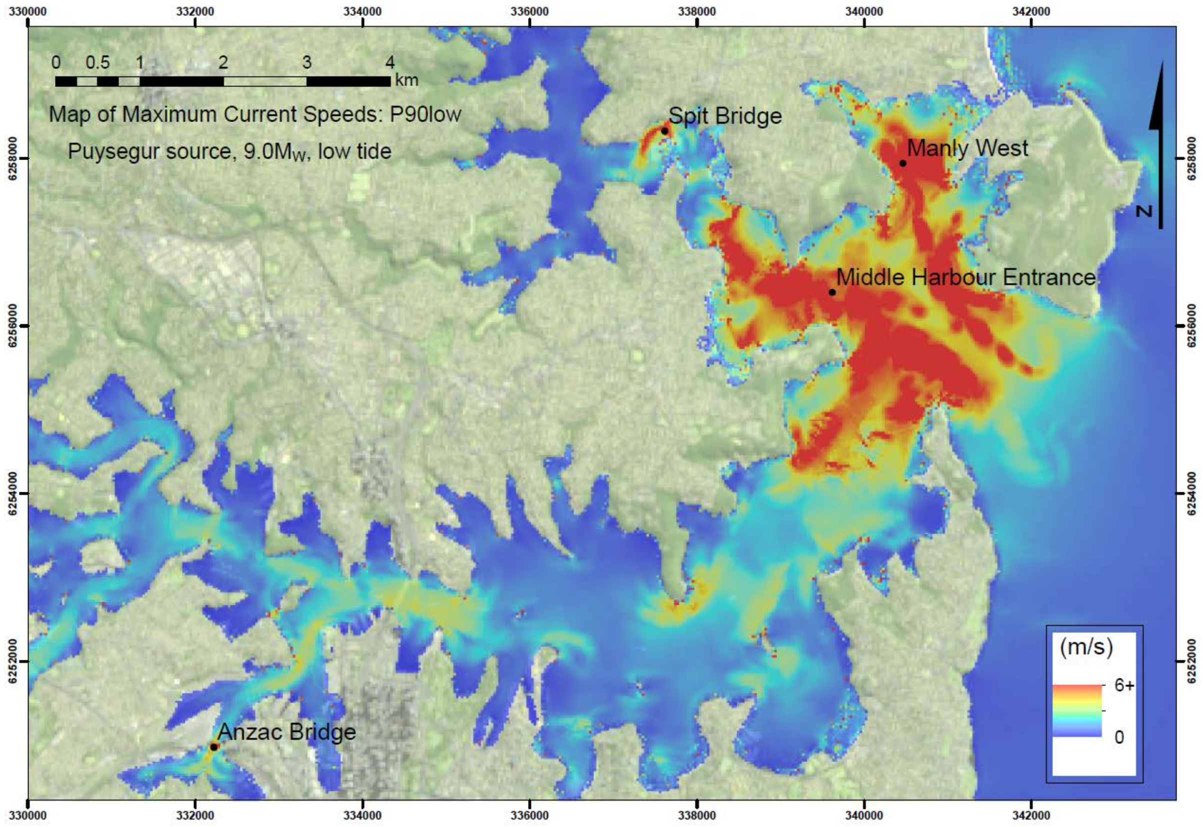

Supplementary Figure S10: Map of maximum current speeds for event NH90high. *This image was created by KMW using ESRI ArcMap 10.3.1 http://www.esri.com/arcgis/about-arcgis, coastline data (https://ecat.ga.gov.au/geonetwork/srv/eng/search#!a05f7892-eae3-7506-e044-00144fdd4fa6) from © Commonwealth of Australia (Geoscience Australia) 2017 and satellite imagery Landsat 8 couresy of the U.S. Geological Survey (https://earthexplorer.usgs.gov/).*

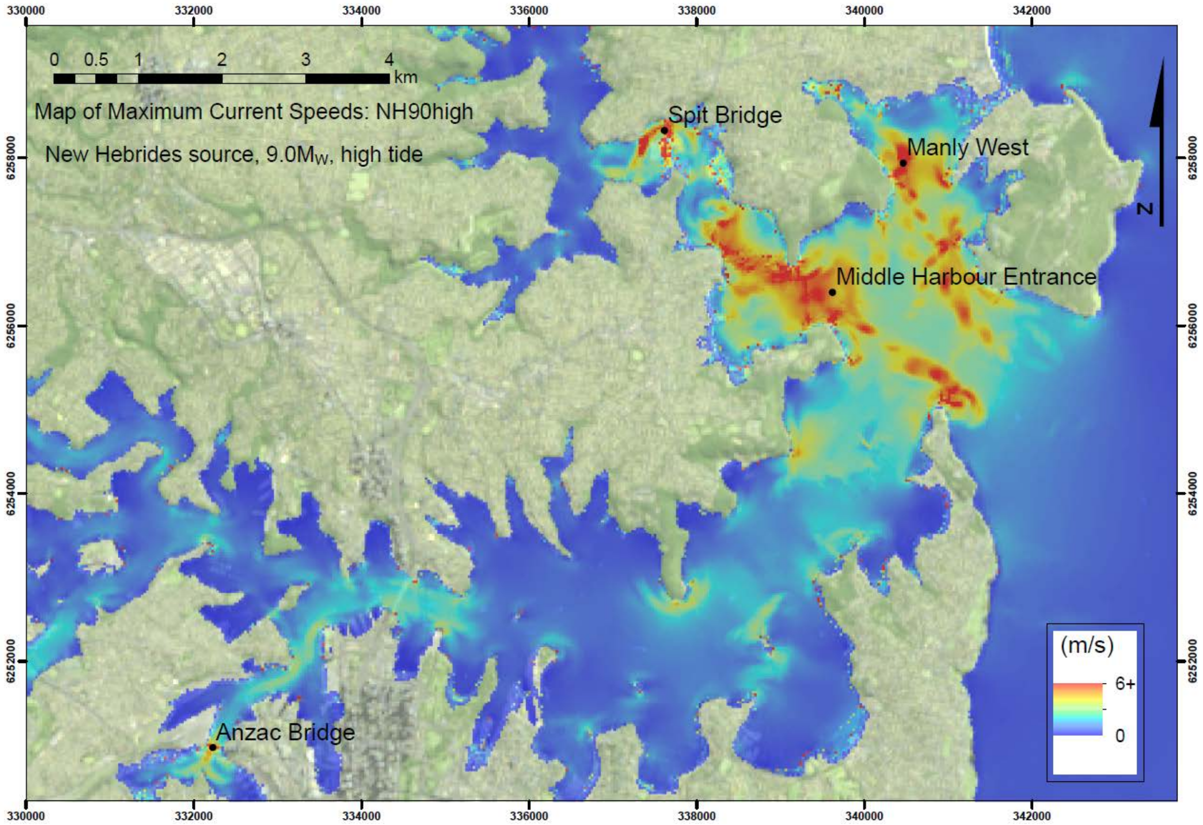

Supplementary Figure S11: Map of maximum current speeds for event NH90low. *This image was created by KMW using ESRI ArcMap 10.3.1 http://www.esri.com/arcgis/about-arcgis, coastline data (https://ecat.ga.gov.au/geonetwork/srv/eng/search#!a05f7892-eae3-7506-e044-00144fdd4fa6) from © Commonwealth of Australia (Geoscience Australia) 2017 and satellite imagery Landsat 8 couresy of the U.S. Geological Survey (https://earthexplorer.usgs.gov/).*

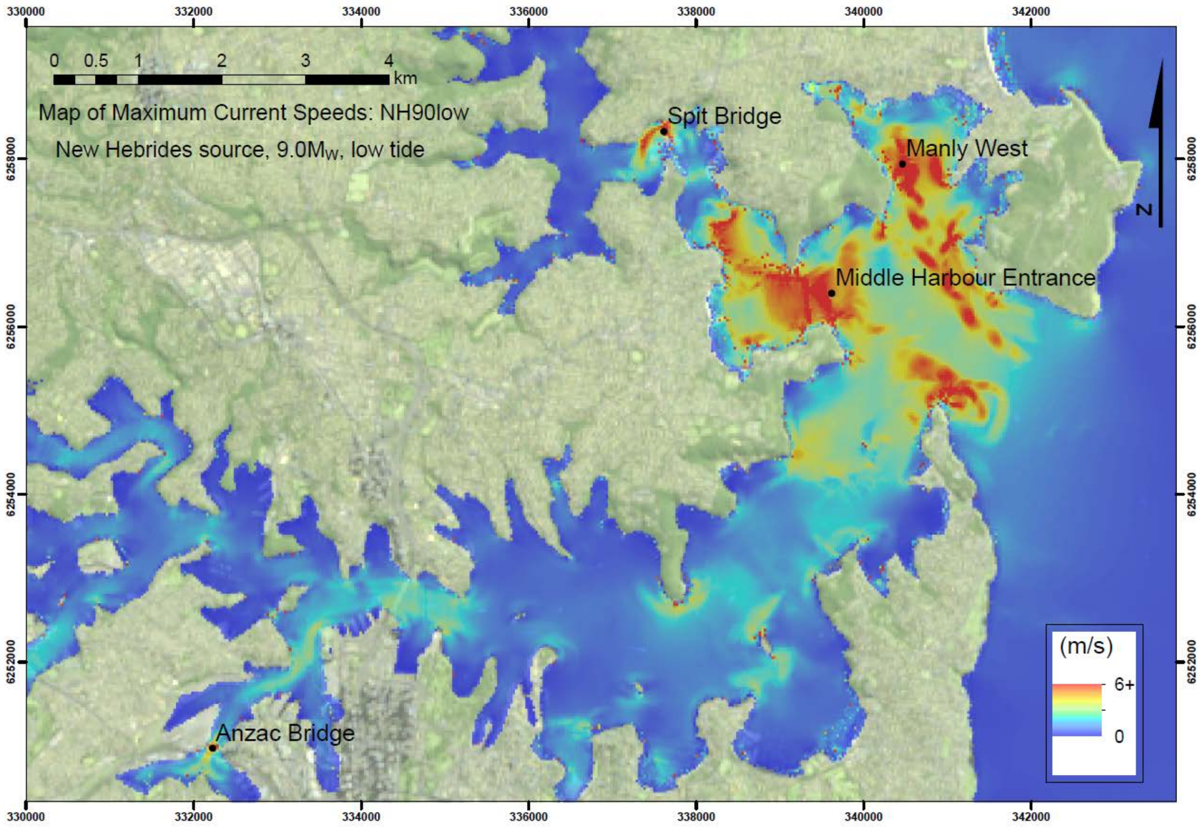

Supplementary Figure S12: Map of maximum current speeds for event P85high. *This image was created by KMW using ESRI ArcMap 10.3.1 http://www.esri.com/arcgis/about-arcgis, coastline data (https://ecat.ga.gov.au/geonetwork/srv/eng/search#!a05f7892-eae3-7506-e044-00144fdd4fa6) from © Commonwealth of Australia (Geoscience Australia) 2017 and satellite imagery Landsat 8 couresy of the U.S. Geological Survey (https://earthexplorer.usgs.gov/).*

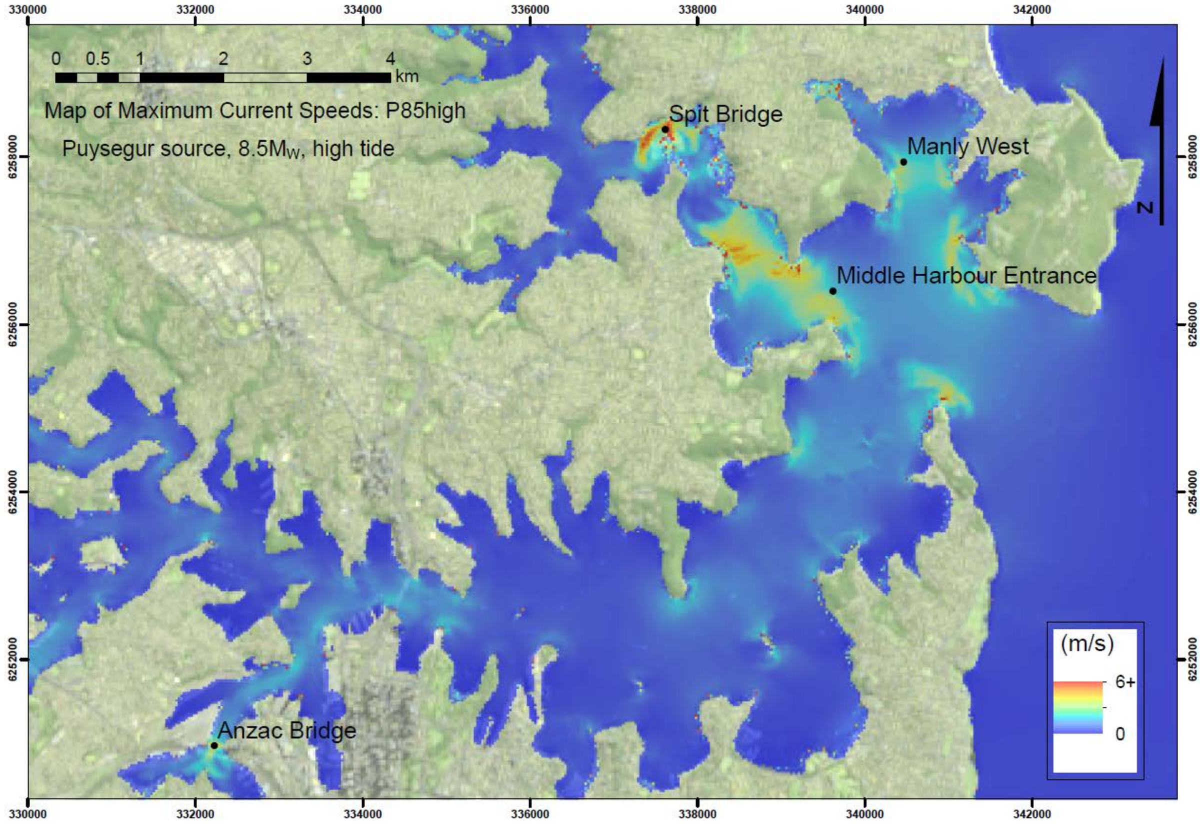

Supplementary Figure S13: Map of maximum current speeds for event P85low. *This image was created by KMW using ESRI ArcMap 10.3.1 http://www.esri.com/arcgis/about-arcgis, coastline data (https://ecat.ga.gov.au/geonetwork/srv/eng/search#!a05f7892-eae3-7506-e044-00144fdd4fa6) from © Commonwealth of Australia (Geoscience Australia) 2017 and satellite imagery Landsat 8 couresy of the U.S. Geological Survey (https://earthexplorer.usgs.gov/).*

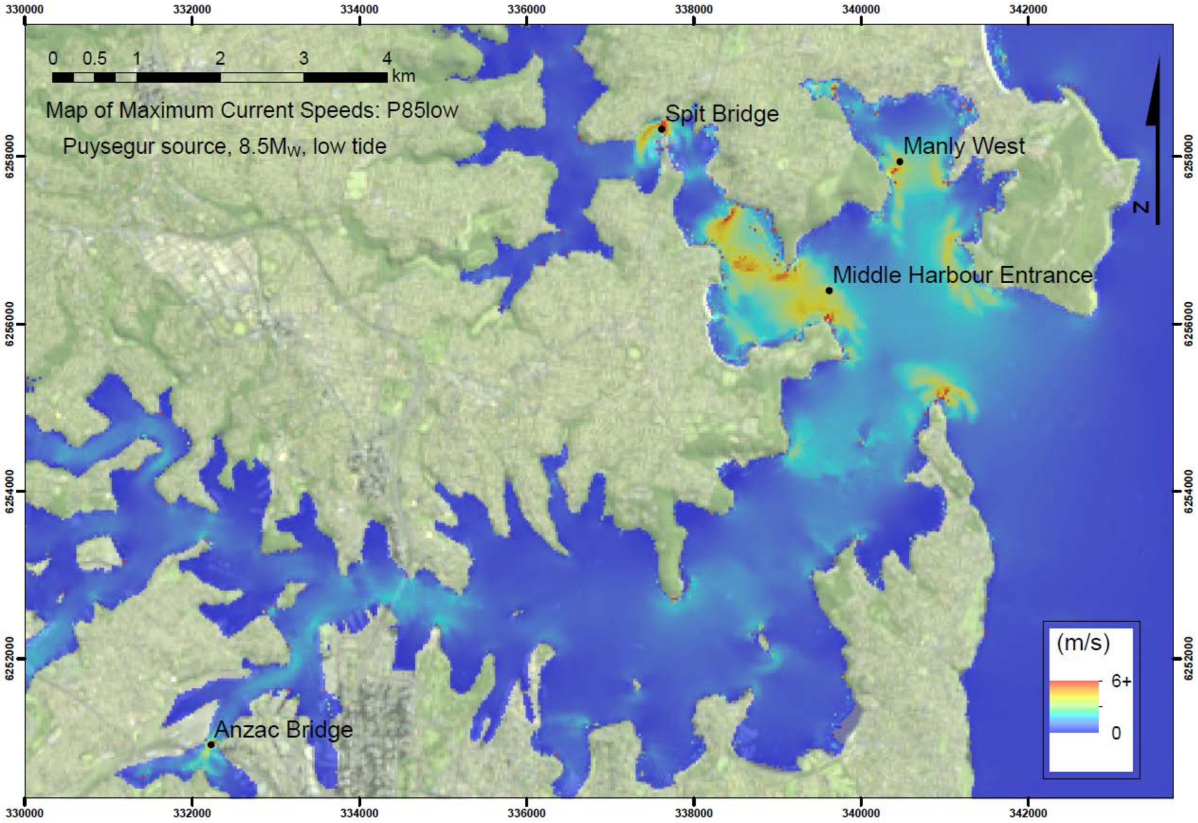

Supplementary Figure S14: Map of maximum current speeds for event NH85high. *This image was created by KMW using ESRI ArcMap 10.3.1 http://www.esri.com/arcgis/about-arcgis, coastline data (https://ecat.ga.gov.au/geonetwork/srv/eng/search#!a05f7892-eae3-7506-e044-00144fdd4fa6) from © Commonwealth of Australia (Geoscience Australia) 2017 and satellite imagery Landsat 8 couresy of the U.S. Geological Survey (https://earthexplorer.usgs.gov/).*

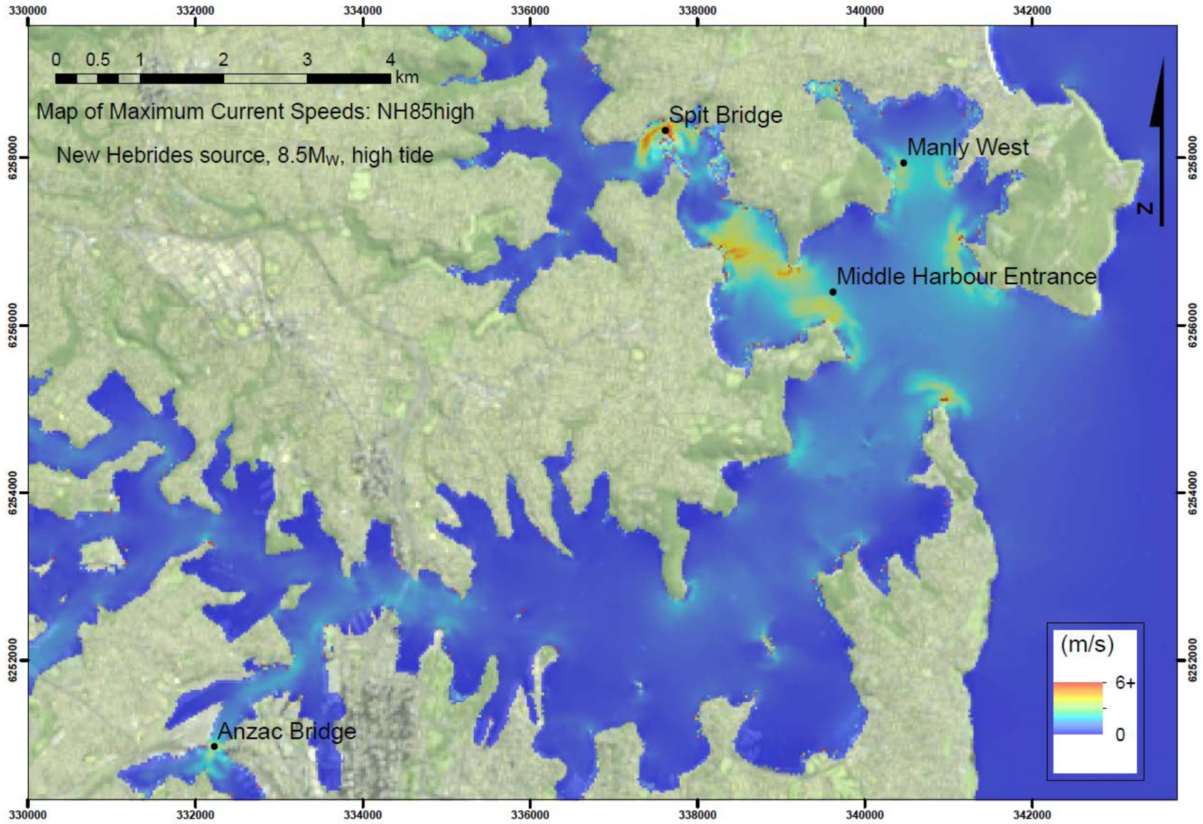

Supplementary Figure S15: Map of maximum current speeds for event NH85low. *This image was created by KMW using ESRI ArcMap 10.3.1 http://www.esri.com/arcgis/about-arcgis, coastline data (https://ecat.ga.gov.au/geonetwork/srv/eng/search#!a05f7892-eae3-7506-e044-00144fdd4fa6) from © Commonwealth of Australia (Geoscience Australia) 2017 and satellite imagery Landsat 8 couresy of the U.S. Geological Survey (https://earthexplorer.usgs.gov/).*

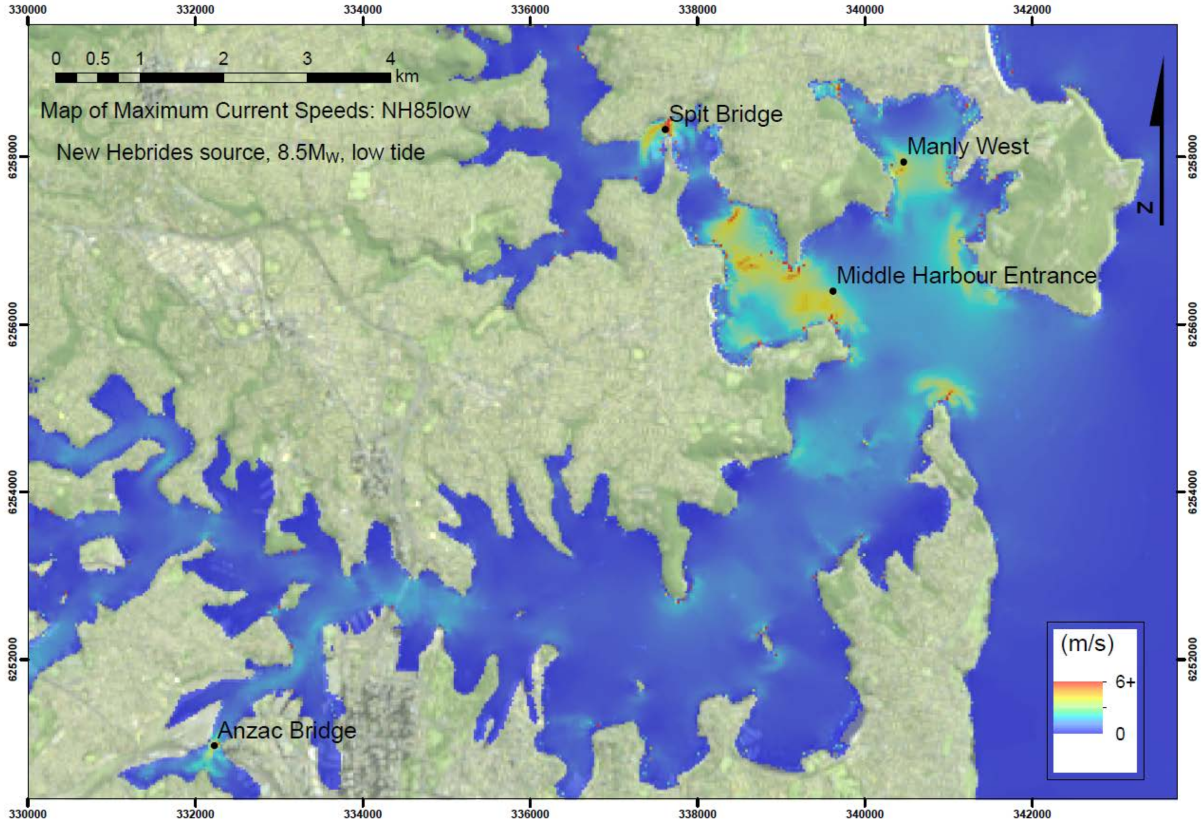

Supplementary Figure S16: Map of maximum current speeds for event Chi1960high. *This image was created by KMW using ESRI ArcMap 10.3.1 http://www.esri.com/arcgis/about-arcgis, coastline data (https://ecat.ga.gov.au/geonetwork/srv/eng/search#!a05f7892-eae3-7506-e044-00144fdd4fa6) from © Commonwealth of Australia (Geoscience Australia) 2017 and satellite imagery Landsat 8 couresy of the U.S. Geological Survey (https://earthexplorer.usgs.gov/).*

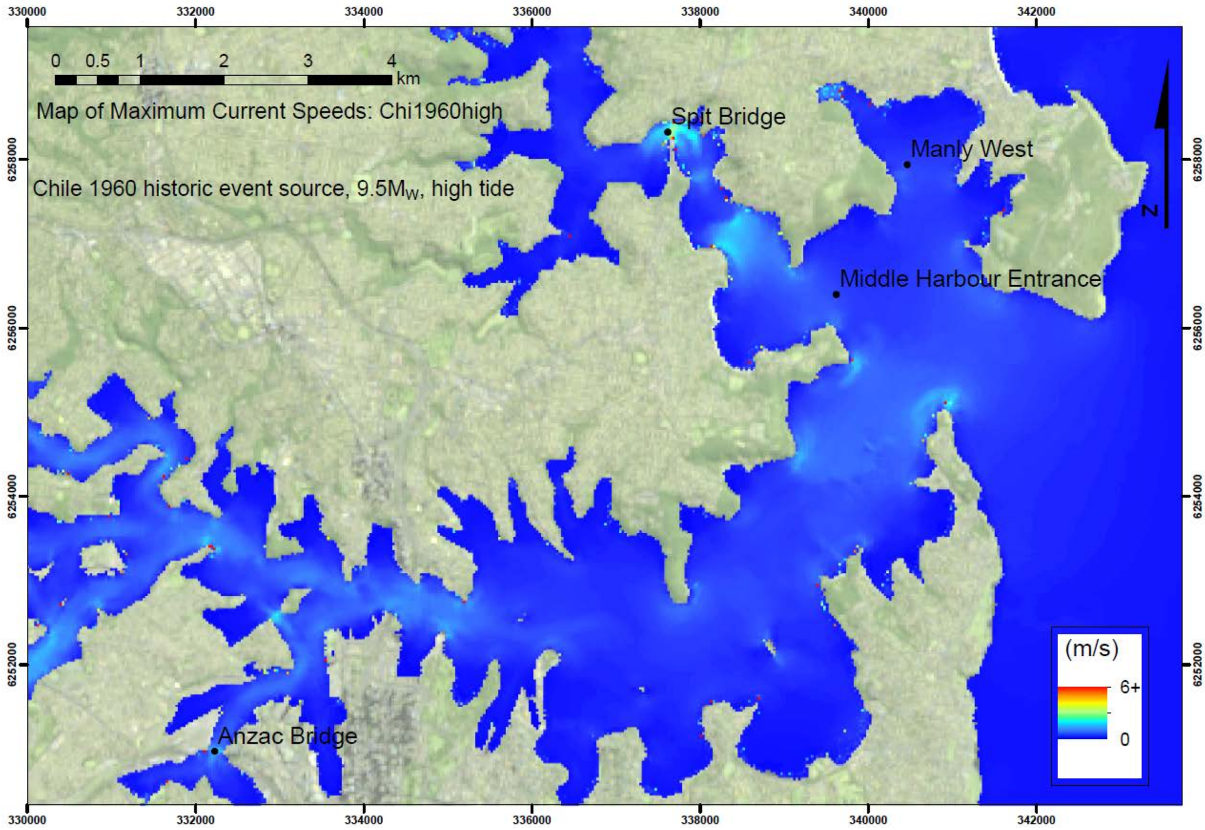

Supplementary Figure S17: Map of maximum current speeds for event Chi1960historic. *This image was created by KMW using ESRI ArcMap 10.3.1 http://www.esri.com/arcgis/about-arcgis, coastline data (https://ecat.ga.gov.au/geonetwork/srv/eng/search#!a05f7892-eae3-7506-e044-00144fdd4fa6) from © Commonwealth of Australia (Geoscience Australia) 2017 and satellite imagery Landsat 8 couresy of the U.S. Geological Survey (https://earthexplorer.usgs.gov/).*

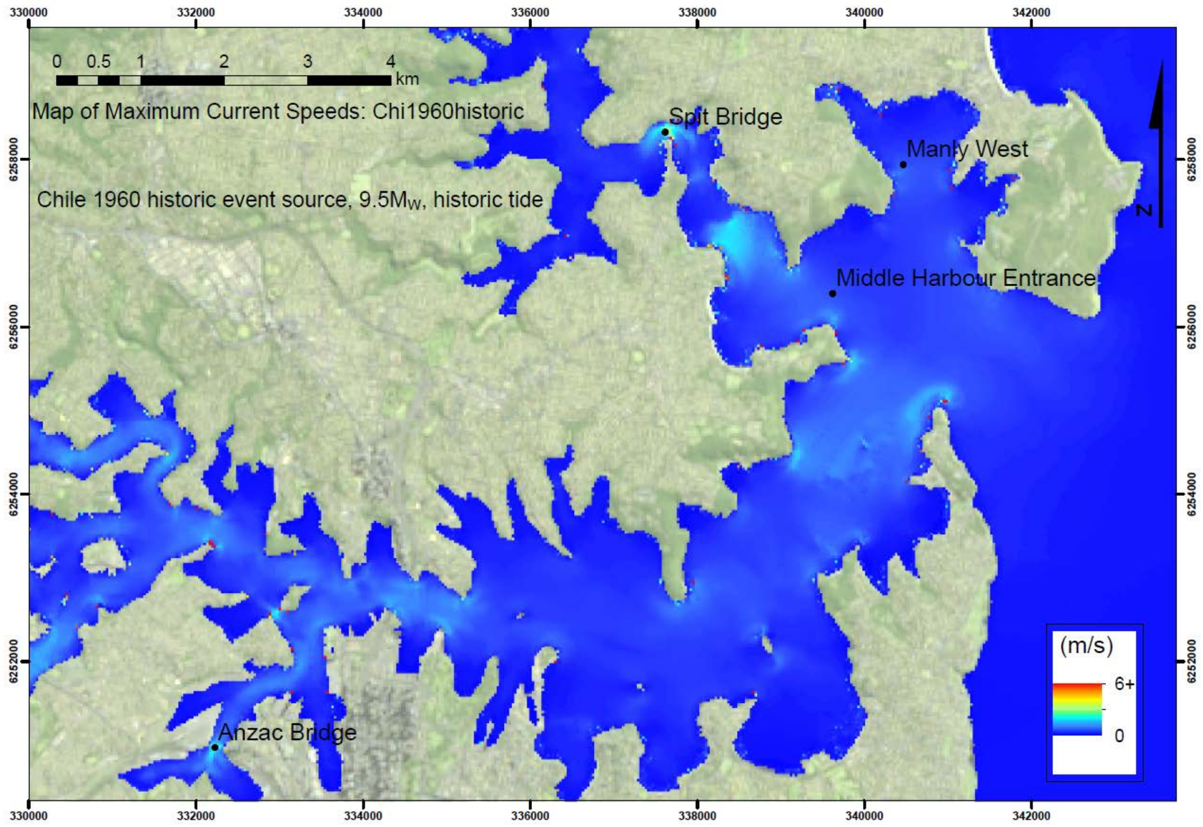

Supplementary Figure S18: Map of maximum current speeds for event Chi1960low. *This image was created by KMW using ESRI ArcMap 10.3.1 http://www.esri.com/arcgis/about-arcgis, coastline data (https://ecat.ga.gov.au/geonetwork/srv/eng/search#!a05f7892-eae3-7506-e044-00144fdd4fa6) from © Commonwealth of Australia (Geoscience Australia) 2017 and satellite imagery Landsat 8 couresy of the U.S. Geological Survey (https://earthexplorer.usgs.gov/).*

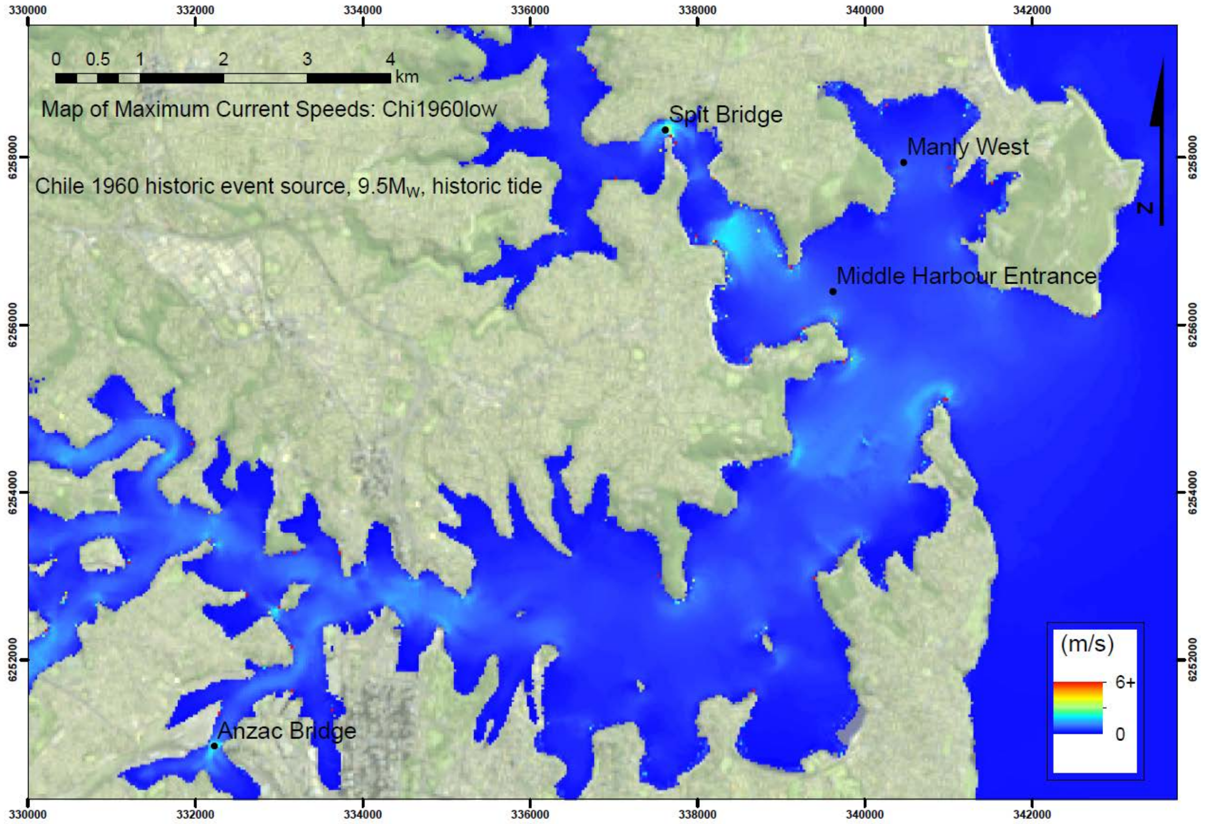

Supplementary Figure S19: Map of maximum current speeds for event Toh2011high. *This image was created by KMW using ESRI ArcMap 10.3.1 http://www.esri.com/arcgis/about-arcgis, coastline data (https://ecat.ga.gov.au/geonetwork/srv/eng/search#!a05f7892-eae3-7506-e044-00144fdd4fa6) from © Commonwealth of Australia (Geoscience Australia) 2017 and satellite imagery Landsat 8 couresy of the U.S. Geological Survey (https://earthexplorer.usgs.gov/).*

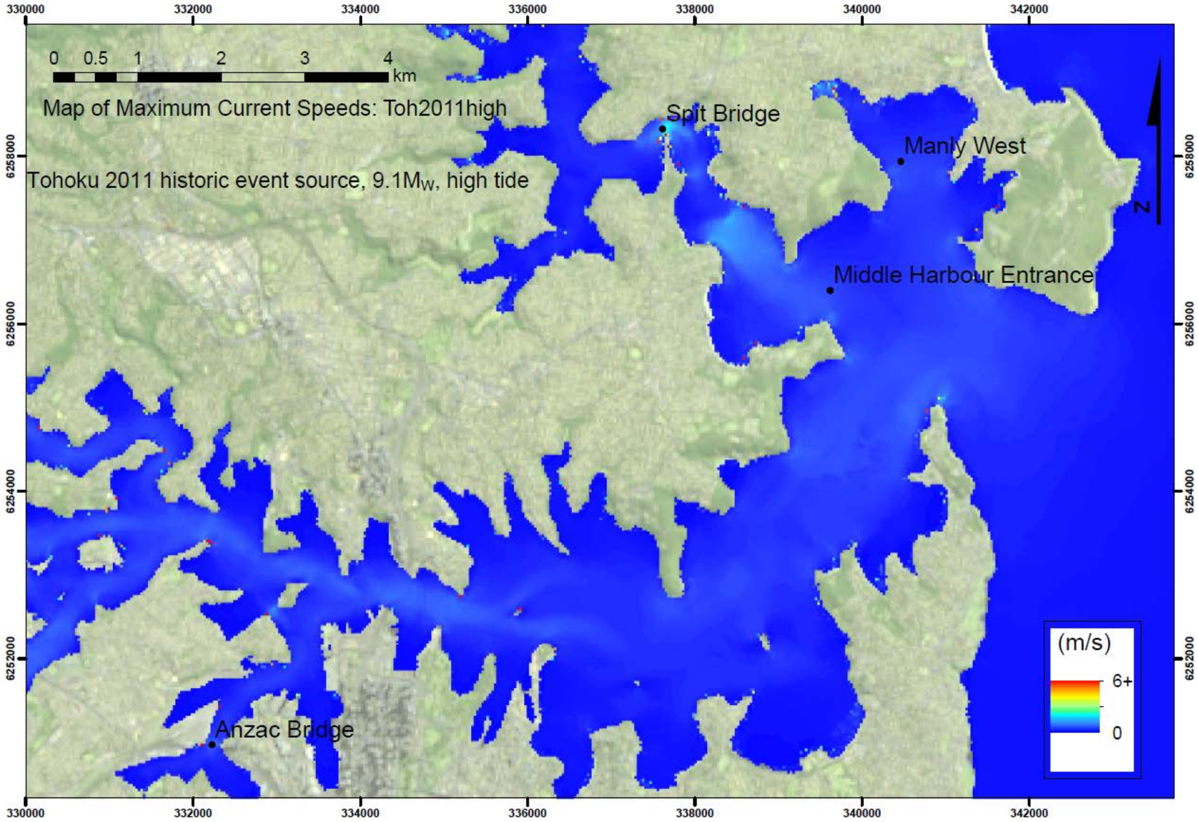

Supplementary Figure S20: Map of maximum current speeds for event Toh2011historic. *This image was created by KMW using ESRI ArcMap 10.3.1 http://www.esri.com/arcgis/about-arcgis, coastline data (https://ecat.ga.gov.au/geonetwork/srv/eng/search#!a05f7892-eae3-7506-e044-00144fdd4fa6) from © Commonwealth of Australia (Geoscience Australia) 2017 and satellite imagery Landsat 8 couresy of the U.S. Geological Survey (https://earthexplorer.usgs.gov/).*

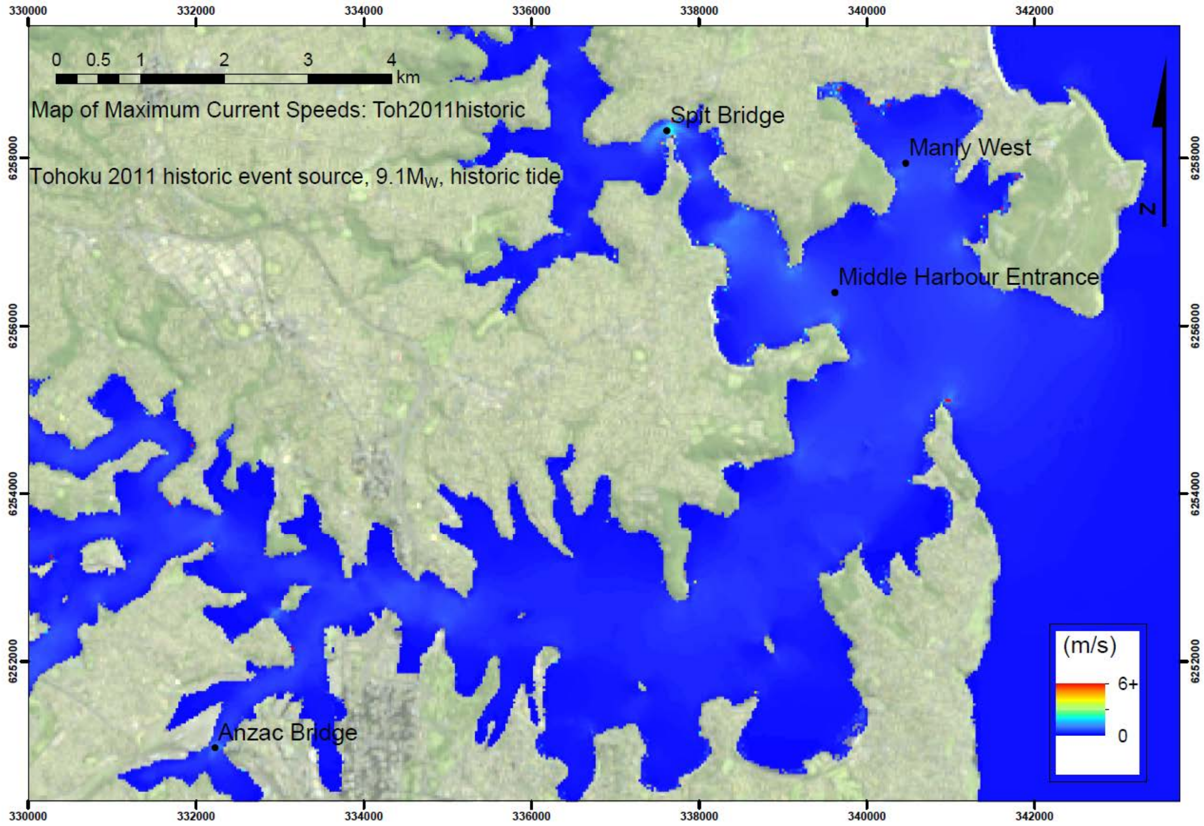

Supplementary Figure S21: Map of maximum current speeds for event Toh2011low. *This image was created by KMW using ESRI ArcMap 10.3.1 http://www.esri.com/arcgis/about-arcgis, coastline data (https://ecat.ga.gov.au/geonetwork/srv/eng/search#!a05f7892-eae3-7506-e044-00144fdd4fa6) from © Commonwealth of Australia (Geoscience Australia) 2017 and satellite imagery Landsat 8 couresy of the U.S. Geological Survey (https://earthexplorer.usgs.gov/).*

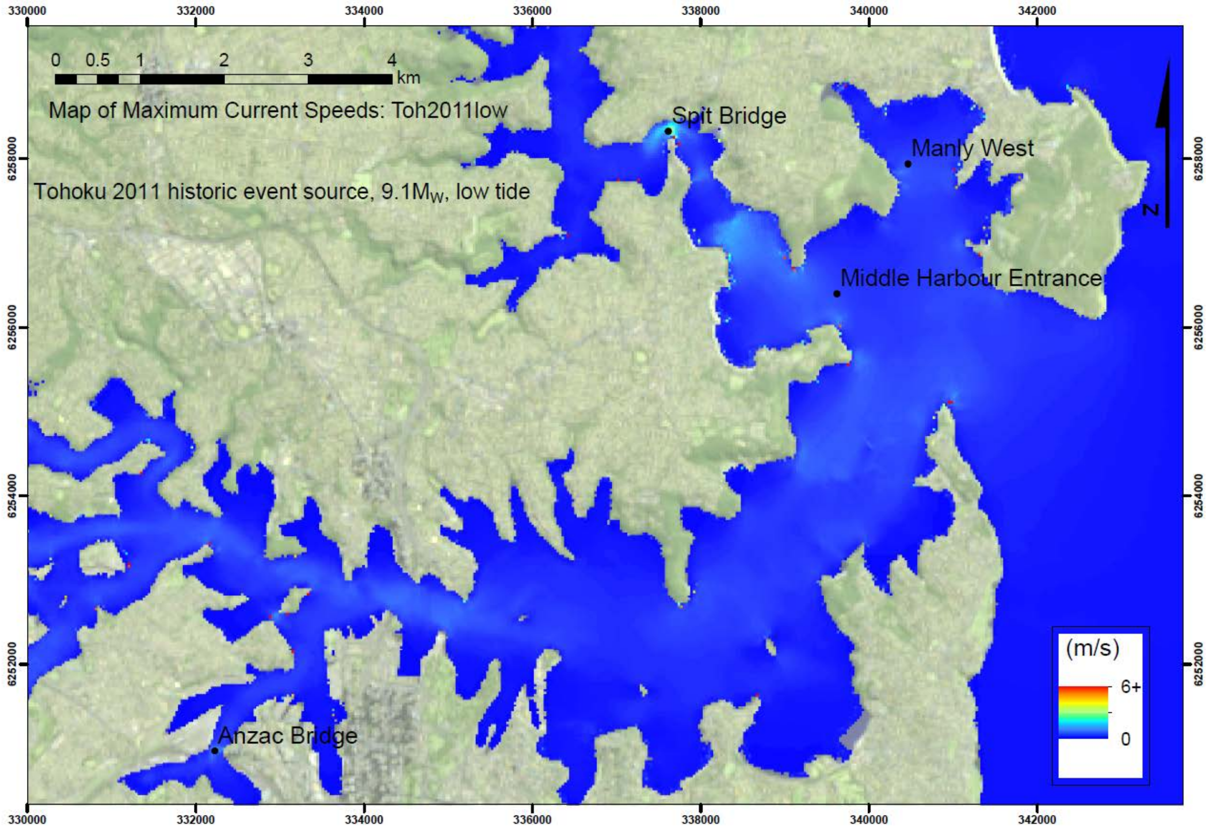

Supplement: Supplementary file 1 — Supplementary Information [file 41598_2018_33156_MOESM1_ESM.pdf]
